# Immobilized lipase enzyme on green synthesized magnetic nanoparticles using *Psidium guava* leaves for dye degradation and antimicrobial activities

**DOI:** 10.1038/s41598-024-58840-y

**Published:** 2024-04-17

**Authors:** Yosri A. Fahim, Ahmed M. El-Khawaga, Reem M. Sallam, Mohamed A. Elsayed, Mohamed Farag Ali Assar

**Affiliations:** 1 Department of Basic Medical Sciences, Faculty of Medicine, Galala University, Galala City, 43511 Suez Egypt; 2 Department of Medical Biochemistry & Molecular Biology, Faculty of Medicine, Ain Shams University, Cairo, 11566 Egypt; 3 Chemical Engineering Department, Military Technical College (MTC), Egyptian Armed Forces, Cairo, Egypt; 4 Department of Chemistry, Biochemistry Division, Faculty of Science, Menoufia University, Shibin El Kom, Menoufia, Egypt

**Keywords:** Enzymes, Nanotechnology, Lipase, Magnetic nanoparticles, Immobilization, Nanoparticles, Chemical engineering, Microbiology

## Abstract

Zinc ferrite nanoparticles (ZnF NPs) were synthesized by a green method using *Psidium guava Leaves* extract and characterized via structural and optical properties. The surface of ZnF NPs was stabilized with citric acid (CA) by a direct addition method to obtain (ZnF-CA NPs), and then lipase (LP) enzyme was immobilized on ZnF-CA NPs to obtain a modified ZnF-CA-LP nanocomposite (NCs). The prepared sample’s photocatalytic activity against Methylene blue dye (MB) was determined. The antioxidant activity of ZnF-CA-LP NCs was measured using 1,1-diphenyl-2-picryl hydrazyl (DPPH) as a source of free radicals. In addition, the antibacterial and antibiofilm capabilities of these substances were investigated by testing them against gram-positive *Staphylococcus aureus* (*S. aureus ATCC *25923) and gram-negative *Escherichia coli* (*E. coli ATCC *25922) bacterial strains. The synthesized ZnF NPs were discovered to be situated at the core of the material, as determined by XRD, HRTEM, and SEM investigations, while the CA and lipase enzymes were coated in this core. The ZnF-CA-LP NCs crystallite size was around 35.0 nm at the (311) plane. Results obtained suggested that 0.01 g of ZnF-CA-LP NCs achieved 96.0% removal of 5.0 ppm of MB at pH 9.0. In-vitro zone of inhibition (ZOI) and minimum inhibitory concentration (MIC) results verified that ZnF-CA-LP NCs exhibited its encouraged antimicrobial activity against *S. aureus* and *E. coli* (20.0 ± 0.512, and 27.0 ± 0.651 mm ZOI, respectively) & (1.25, and 0.625 μg/ml MIC, respectively). ZnF-CA-LP NPs showed antibiofilm percentage against *S. aureus* (88.4%) and *E. coli* (96.6%). Hence, ZnF-CA-LP NCs are promising for potential applications in environmental and biomedical uses.

## Introduction

The utilization of green synthesis methods for nanoparticles offers several advantages compared to traditional physical and chemical procedures, based on the abundance of biological entities and the use of ecologically friendly technologies^1^. The green synthesis of nanoparticles by different macroscopic or microscopic organisms, or their derivatives, such as plant extracts, bacteria, fungi, yeast, and microalgae has recently gained an enormous interest^2^. For instance, the utilization of plants in green technology is becoming increasingly popular as an environmentally benign, non-toxic, secure, and economically advantageous alternative. Employing plant extracts to synthesize nanoparticles also provides natural components for capping agents^3^. The primary objective of green synthesis research is to enhance a synthesis technique for creating environmentally friendly, non-toxic, superparamagnetic, and cost-effective magnetic nanoparticles^5^. The synthesis and characterization of magnetic nanoparticles, which are simply defined as particles with dimensions on the nanoscale that can react to magnetic fields, are increasingly attracting attention due to their exceptional capabilities in diverse applications. Due to their magnetic properties and the fact that they are simple and safe to synthesize in a laboratory, magnetic nanoparticles have promising applications in various industries^4^. The following section explains the current work approach of using zinc ferrite-magnetic nanoparticle particles to immobilize lipase enzymes. Magnetic nanocatalyst has the advantageous capability of being efficiently separated from the reaction medium through the use of an external magnet, hence obviating the necessity for additional filtration, centrifugation, or other laborious techniques^5^. Lipases, also known as triacylglycerol acyl hydrolases (EC 3.1.1.3), belong to the hydrolases family of enzymes specialized in catalyzing the hydrolysis of triacylglycerol (TAG) ester linkages, producing free fatty acids (FFAs) and glycerol. They are important biocatalysts with exceptional selectivity enabling them to be utilized in a variety of processes, including the production of pharmaceuticals and biofuels^6^. Their ability to catalyze specific ester degradation under ambient circumstances without generating undesirable byproducts allows them to play a critical role in the global market. Therefore, scientists continue to investigate their potential applications in a variety of disciplines, ranging from medicine and biotechnology to environmental sciences and renewable energy^7^. Despite diverse applications of lipases, serious limitations for their industrial applications exist. This is based on their weak stability, ease of inactivation, and difficulty in separating from the reaction system for repurposing^8^. Therefore, lipase activity and operational stability must be enhanced by a wise choice of immobilization procedure to attain more affordable and effective use in reaction systems^9^. Immobilization of enzymes is a well-established technique that enables their application in a variety of bio-catalyzed processes. This method has several benefits, including better product recovery, the ability to reuse the biocatalyst, and the frequent improvement of the enzyme's resistance to denaturants, resulting in more stable catalysts^10^.

Treatment of antibiotic-resistant organisms has limitations and downsides, such as a rise in hospital infections brought on by these pathogens and difficulties recruiting people for clinical studies assessing novel therapies^11^. Continuous abuse of antimicrobial medications has resulted in the development of resistant and challenging-to-treat infectious infections, while there hasn't been much progress in the discovery of new antibiotics^12^. Antibiotic resistance can be partially addressed by increasing dosage, but eventually, the necessary dose becomes hazardous, costly, or impractical, rendering the illness incurable^13^. It is difficult to assess novel treatments for resistant bacteria due to the absence of data on crucial patient features, advancements in medical practice, and the emergence of antibiotic resistance^14^. Thus, it is advised to assess new medicines through randomized clinical trials, and creative trial designs are being created to reduce the difficulty of recruiting participants while assessing the advantages and disadvantages of novel treatments. Because of their distinct modes of action and antibacterial qualities, nanoparticles can be utilized to treat pathogenic bacteria in place of antibiotics. Treatments based on nanomaterials, including metal oxide nanoparticles can target hard-to-treat biofilms and get around mechanisms of antibiotic resistance^15^. Metal and metal oxide nanoparticles have shown promising antibacterial activity, making them the ideal nanomaterials for biological applications as listed in Table 1.

**Table 1:** Different green synthesized metal oxide NPs and their applications.

Metal oxide NPs	Applications	References
ZnO	Bactericidal activity against skin ulcer pathogens Antifungal and antibacterial agents in agriculture Anticancer activity Photodegradation of methylene blue dye	^16^ ^17^ ^18^ ^19^
CuO	Wound dressing application	^20^
TiO2	Eco-friendly bactericidal agents with wound-healing properties	^21^
ZnO/CuO	Wound healing management Antimicrobial capability against non-MDR and MDR skin pathogens	^22^ ^23^
Al_2_O_3_	Water treatment and biomedical applications	^24^
NiO	Antimicrobial and anticancer activity Photocatalytic activity for degradation of polyethylene film	^25^ ^26^

Nanomaterials are employed as antimicrobial agents due to their ability to undertake several mechanisms within bacterial cells such as causing damage to the bacterial cell membrane; 2) producing reactive oxygen species (ROS); 3) entering the bacterial cell membrane; and 4) triggering intracellular antibacterial effects, such as interactions with DNA and proteins^27–29^, in contrast to conventional antibiotics that mostly rely on singular structures or processes. Nanomaterials' antimicrobial action is influenced by several factors including their dimensions, surface area, chemical composition, and propensity for agglomeration^30^. The behavior of nanomaterials is influenced by the proportion of atoms and molecules present on their surface^31^. One potential application of nanoparticles is their utilization as drug carriers, owing to their diminutive size and potential biocompatibility, provided that their surface electron configuration, which plays a role in cytotoxicity, is appropriately modified^32^. Enzymes have great biocompatibility and possess a high degree of catalytic efficiency; they are known to possess both antiviral and antibacterial capabilities. Enzyme immobilization into nanoparticles enhances overall antibacterial activity, resulting in a synergistic effect ^33^. The synergistic effect of incorporating metal nanoparticles with the matrix-degrading enzyme boosts the efficacy toward pathogenic bacteria^34^. A further potential application for the enzyme immobilized on magnetic nanoparticles is in the field of environmental purification from pollutants. Specifically, wastewater treatment by removal of cationic dyes from water which has gathered significant attention among researchers due to the potential detrimental effects they may have on ecosystems and the overall water quality. The current state of global water resources is experiencing a decline across all nations and is calling for cost-effective and relatively simple interventions^35^.

*Psidium guava* is a medicinal plant, popularly known as guava^36^. It is found across the tropical and subtropical zones and has been proven to have medicinal properties in antibacterial applications^37^, antidiarrheal^38^, antispasmodic^39^, antioxidant ^40^, antiallergy^41^, antibacterial^42^, anti-cough^43^, anti-inflammatory^44^ and anticancer effects^45^. Guava leaves include phytochemicals such as phenol, tannin, terpene, saponin, and flavonoids, which aid in their antibacterial capabilities^46^. Young and mature leaf extracts have been shown to inhibit both Gram-positive (*Staphylococcus aureus* and *Bacillus cereus*) and Gram-negative bacteria (*Salmonella enterica*)^47^.

The objective of the current research is the green synthesis and characterization of zinc ferrite NPs using *Psidium Guava* leaves extract followed by citric acid functionalization and then immobilization of lipase enzyme to get the end nanocomposite (ZnF-CA-LP). The further objective includes investigating the potential application of the synthesized nanoparticles as photocatalysts for methylene blue degradation and also as antibacterial and antibiofilm agents for Gram-negative (*E. coli*), and Gram-positive (*S. aureus*) bacteria.

## Materials and methods

### Materials

Materials used and their commercial sources are as follows: Anhydrous Ferric chloride FeCl_3_; 96% (ADVENT Chem Bio), Zinc chloride ZnCl_2_; 98% (ADVENT Chem Bio), Sodium hydroxide (Alpha Chemika), p-Nitrophenyl Palmitate (PNPP); (Thermo Fisher Scientific), Aspergillus niger lipase (LOBA Chemie, India), Tris buffer superior extra pure 99.9% (Srichem), p-Nitro phenol (MERCK), Isopropyl alcohol 99% (Alpha Chemika), 1,1-diphenyl-2-picryl hydrazyl (DPPH) (Sigma Aldrich), Hydrochloric acid (Alpha Chemika), Methylene Blue dye (Alpha Chemika), Crystal violet (Alpha Chemika), Sodium Chloride (Alpha Chemika), Ethanol 99% (Alpha Chemika) and Gentamycin (GEN) Antibiotic disc (Oxoid). All the chemicals utilized were of reagent grade and employed without undergoing additional purification. All experiments were conducted using Ultrapure Milli-Q water.

### Methods

#### Preparation of *Psidium guava* leaves extract

Fresh leaves of *Psidium guava* were purchased from the local market in Cairo City, Egypt., followed by a thorough washing process using water to eliminate any impurities. Subsequently, the leaves were rinsed with distilled water to eliminate any contaminants. The rinsed leaves were placed within a Soxhlet apparatus and thereafter allowed to cool to ambient temperature. The solution undergoes filtration and subsequent centrifugation at a speed of 4000 (rpm) for 5 min^48^. The plant extract that has been acquired is freshly prepared for immediate utilization and can also be preserved at 4C^0^ for 2–3 days, if necessary^49^.

#### Green synthesis of Magnetic Zinc ferrite (ZnFe_2_O_4_)

The utilization of *Psidium guava* leaf extract was employed in the synthesis of magnetic ZnFe_2_O_4_ (ZnF) nanoparticles, serving as both a reducing agent and a capping medium. Two different solutions were prepared, consisting of 2M FeCl_3_ and 1M ZnCl_2_, respectively. Subsequently, the components were combined within a flask with continuous agitation of the solution. The pH was adjusted to a value of 12 by gradually adding appropriate quantities of 1M of sodium hydroxide solution. Then a steady addition of leaf extract was implemented at a rate of 5 ml/min. The resulting mixture was subjected to continuous stirring for 15 min at a temperature of 65 °C^50^. This process yielded a black precipitate composed of ZnF NPs. The particles acquired were black and demonstrated a pronounced magnetic reactivity. The material underwent a triple-washing process using ethanol and distilled water. The precipitates obtained were subjected to oven drying at a temperature of 60 °C, followed by calcination at 400 °C for 4 h^51^. These dried and calcinated precipitates were then kept for future utilization.

#### Citric acid-coated Zinc Ferrite magnetic nanoparticle (ZnF-CA)

Citric acid (CA) possesses the capacity to act as a stabilizer to prevent the agglomeration of particles and enhance the dispersion of nanoparticles within a solution. The ZnF NPs underwent surface modification through the direct addition of CA, resulting in the formation of modified ZnF NPs containing carboxylic groups^52^. In this experiment, ZnF NPs were combined with CA solution in water (0.02 g/ml) at a temperature of 60 °C. The mixture was stirred for 90 min. The black precipitate underwent multiple washes and was subsequently re-suspended in water with a pH level approximating neutrality, namely within the range of 7 to 7.4. The pH range used to investigate the adsorption of CA onto the magnetite surface was between 4.58 to 7.08. A solution of sodium hydroxide (1M) was employed to get a suspension pH in proximity to 7.0^53^.

#### Immobilization of lipase enzyme on ZnF-CA nanocomposite (ZnF-CA-LP) NCs

The immobilization process involved the utilization of a 30 mg mL^-1^ lipase solution in a 50 mM Tris–HCl buffer at pH 8, with the addition of 200 mg of ZnF-CA NCs. The reaction was conducted with continuous stirring at 3000 rpm for one hour at room temperature^54^.

### Characterization techniques of the prepared samples

#### UV–Vis spectroscopy

The UV–Vis absorption spectra of magnetic nanoparticles were measured using a UV–vis spectrophotometer (Agilent Technologies Cary 60 UV–vis) throughout the wavelength range of 200–800 nm. The samples underwent dilution and dispersion in double-distilled water under ambient conditions.

#### X-ray diffraction (XRD)

The X-ray diffraction patterns were conducted using XRD-6000, Shimadzu apparatus, SSI, Japan. The radiation source employed was CuKα (40 kV, 40 mA), and a secondary beam graphite monochromator was utilized. The recorded patterns encompassed a 2-theta (2θ) range spanning from 10 to 80°, with increments of 0.02° and a counting duration of 2 s per step.

#### Fourier-transform infrared spectroscopy (FTIR)

The investigation of the functionalized surface of green ZnF nanoparticles and the confirmation of lipase immobilization were conducted by Fourier-Transform Infrared (FTIR) spectroscopy. This analysis was performed using an FTIR 4700 spectrometer (Jasco, Tokyo, Japan) fitted with a Peltier stabilizer DLaTGS detector. The specimens were produced using potassium bromide (KBr) pellets. The FTIR spectra were obtained within the frequency range of 400–4000 cm^−1^, with a resolution of 4 cm^−1^ and an average of 32 scans.

#### Scanning electron microscopy (SEM)

Scanning electron microscopy (SEM) is a rapid and accurate method for examining the surface morphology and microstructures of a material. SEM pictures and energy-dispersive X-ray spectroscopy (EDX) data were acquired using TESCAN VEGA COMPACT SEM with Tungsten Filament as electron source and attached EDX detector JEOL JSM-6510 LV SEM Microscope (Kohoutovice, Czech Republic). The SEM was operated at a voltage of 10 kV.

#### Transmission electron microscopy (TEM)

The study of the shape and average size of synthesized nanoparticles was conducted using a High-resolution transmission electron microscopy (HRTEM, JEOL 3010, Japan) operated at 300 kV. The nanoparticles were evenly distributed in ethanol and applied onto a copper grid with a lacy structure using a drop-casting technique. Subsequently, the sample was allowed to dry for one hour before being subjected to examination.

#### Vibrating sample magnetization (VSM)

The magnetic attitude of Magnetic nanoparticles was investigated using VSM (Lakeshore 7410, USA) with an applied field from 0 Oe to 20,000 Oe at room temperature. In terms of the magnetization curve of the synthesized magnetic nanoparticles, the hysteresis loop with measurable remanence and coercivity values was observed.

### Antioxidant activity of magnetic ZnF-CA-LP NPs

The antioxidant activity was conducted using D1,1-diphenyl-2-picryl hydrazyl (DPPH) technique. 20 mg of ZnF-CA-LP NCs was evenly dispersed within a glass vial that already contained 1.3 mL of DPPH solution (with a concentration of 100 µmol/L in methanol)^55^. The DPPH radical exhibits a distinct violet color when dissolved in a solution, and over time, it transitions to a colorless or faint yellow shade in the presence of ZnF-CA-LP NCs. This characteristic enables convenient observation and tracking of the reaction. A negative control sample consisting of DPPH in methanol without the inclusion of the powder samples was maintained, while ascorbic acid was used as a positive control or standard for comparison testing nanoparticles. The interaction at the surface between ZnF-CA-LP NCs and DPPH reagent was facilitated by the application of gentle magnetic stirring. The resulting supernatant after centrifugation was collected at 15-min intervals for analysis using UV–VIS spectroscopy at 517 nm against the methanol as blank. Each sample was analyzed in triplicate^56^. The percentage of inhibition was calculated against blank:

1 $${\text{DPPH}}\;{\text{radical}}\;{\text{scavenging}}\;\% = \left[ {\left( {{\text{A}}0 - {\text{A}}1} \right)/{\text{A}}0} \right] \times 100$$

where A_0_ is the absorbance of the DPPH solution and A_1_ is the absorbance of the sample at specific time.

### Photocatalytic activity measurement

In this experimental study, a total of 10 mg of several types of nanoparticles (ZnF NPs, ZnF-CA NPs, and ZnF-CA-LP NCs were solubilized in a 50 ml aqueous solution containing Methylene blue (MB) with an initial concentration of 10.0 ppm. The solubilization process was carried out under continuous stirring. Then, the mixture was placed in a light-restricted chamber for 30 min to establish a state of equilibrium between the processes of adsorption and desorption of the photocatalyst and methylene blue dye. After the completion of the solution, it is subjected to irradiation using a UV lamp. At regular intervals of 15 min, a volume of 2.0 ml of the sample was extracted from the tube and subjected to centrifugation to facilitate the separation of the photocatalyst. The absorbance of the solution was measured at a wavelength of 664.0 nm using a UV–Visible spectrophotometer^57^. The proportion of degradation was determined by the utilization of a mathematical equation.

2 $$\mathrm{Percentage \;of \;degradation }=\frac{Ci-Cf}{Ci}{\text{X}}100$$

where C_i_ represents the initial absorption of the dye and C_f_ represents its absorption following a different time (min). The identical operation is conducted using variables such as pH, dye concentration, catalytic dose, and presence of H_2_O_2_.

### Reusability of Magnetic ZnF-CA-LP NCs

The reusability of ZnF-CA-LP NCs was assessed by conducting multiple consecutive operation cycles for photocatalytic reduction of MB dye when subjected to UV irradiation for 120 min. After every 120 min, a sample of the dye solution was withdrawn and analyzed for residual dye concentration by the UV–Vis spectrophotometer, and the Nanocomposite was separated with the magnet. The ZnF-CA-LP NCs were thoroughly rinsed with water. Subsequently, its reusability was used by putting it into another MB solution to initiate a fresh cycle. The ZnF-CA-LP NCs activity was quantified in each cycle and expressed as a relative value.

### Antimicrobial activity and minimal inhibitory concentration (MIC)

Both the minimum inhibitory concentration (MIC) and the disk diffusion method on agar were employed in the antibacterial studies. The study involved the examination of two separate strains, specifically one strain classified as gram-positive (*S. aureus ATCC25923*), and another strain classified as gram-negative (*E. coli ATCC25922*). The broth solution was supplied with each strain at a concentration of 0.5 MacFarland for the subsequent dilution operation. The method employed to ascertain the lowest concentration required to hinder the proliferation of microorganisms was referred to as the MIC^58^.

To investigate the effects of different nanoparticle concentrations in Mueller–Hinton (MH) broth, a series of experiments were conducted in triplicate. These experiments were carried out using 96-well plates^59^. A study was done wherein three various types of inhibition controls were employed. The positive inhibition control consisted of Gentamycin at a concentration of 10 µg/ml. The negative control was established by DMSO. The microplates were subjected to an incubation period lasting 48 h, during which a consistent temperature of 37 °C was maintained. The optical density at 600 nm was measured using a primary filter and a microplate spectrophotometer^60^.

The Kirby-Bauer method was implemented during the disc diffusion examination. The microorganism inoculum was evenly distributed across the entire surface of the Mueller–Hinton agar in a petri dish^61^. Subsequently, a 10 µg/ml nanoparticle layer was evenly distributed across the surface of the agar. The containers were subjected to an incubation period of 24 h at a temperature of 37 °C. Whether or not the discs were encircled by a halo of growth inhibition was utilized to assess the outcomes.

### Antibiofilm activity of the synthesized ZnF-CA-LP NCs

Biofilms, composed of polysaccharides, proteins, and nucleic acids, serve as barrier systems against pathogens and infections^62^. A qualitative evaluation of biofilm formation was performed by visually examining the biofilm that developed on the inner surface of the tube^63^:. The antibiofilm efficacy of the ZnF-CA-LP NCs of a concentration of 10 µg/ml was evaluated with a control sample, with a specific focus on susceptible bacterial strains without the presence of ZnF-CA-LP NCs and with the presence of ZnF-CA-LP NCs. A quantity of five milliliters of nutrient broth was inserted into the tubes, subsequently followed by the introduction of an aliquot containing 0.5 McFarland of the bacteria being studied^64^. The tubes were thereafter placed in an incubator set at a temperature of 37 ^0^C for a duration of 24 h.

The contents of the treated and untreated containers were extracted and subsequently submitted to treatment with Phosphate Buffer Saline (PBS) at a pH = 7, followed by dehydration. The bacterial layers were rendered stable for 10 min by treating them with a solution containing 3.0% sodium acetate (5 ml), Subsequently, they were rinsed using deionized water.

The biofilms were subjected to a staining procedure utilizing a 0.1% concentration of Crystal Violet (CV) for 15 min. Following that, the biofilms underwent a washing procedure using deionized water to remove any remaining discoloration. To promote the extraction of the stain, a volume of 2.0 ml of ethanol was combined. The observation of a visible pigmented coating on both the upper and lower surfaces of the tube indicated the formation of a biofilm with a favorable result. The quantification of bacterial biofilms was performed using a UV–Vis spectrophotometer, which was adjusted at a wavelength of 580.0 nm. The determination of the percentage of inhibition was carried out using the designated equation^65^.

3 $$Bacterialbiofilm\mathrm{ \;inhibition }\left(\mathrm{\%}\right) =\frac{O.D.\mathrm{of \;control }\;sample-O.D.\mathrm{of \;treated \;sample }}{O.D.of\;control\;sample}\mathrm{ X }100$$

### Statistical analysis

The statistical analysis of our results was conducted utilizing the ONE-WAY ANOVA (with a significance level of (*P* < 0.05). Specifically, we employed Duncan's multiple range test and the least significant difference (LSD) method for summarizing the findings. The investigation and evaluation of the outcomes and data were conducted using SPSS version 15. Data were processed in the Origin Pro 8.5 SR1 software.

## Results and discussion

### Characterization of magnetic nanoparticles

#### UV–Vis spectroscopy analysis

**Figure 1:**
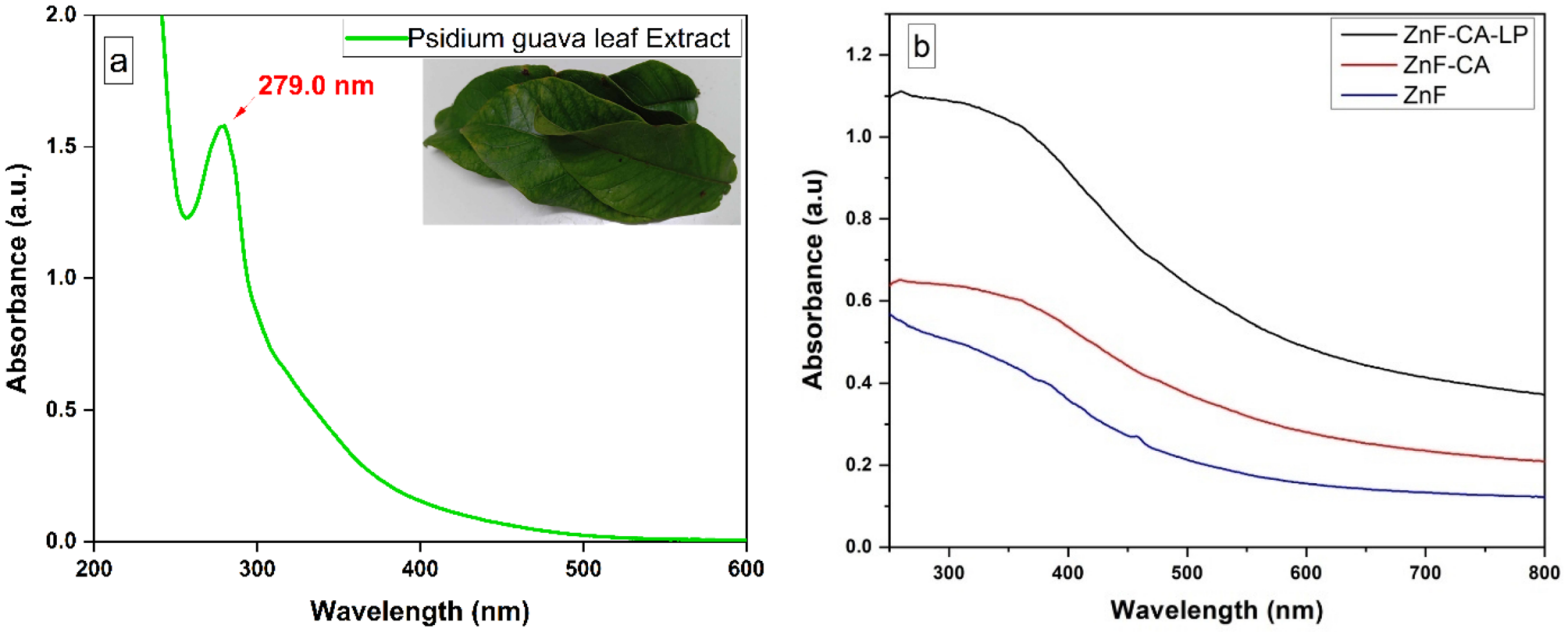
UV spectrum of (**a**) Psidium guava leaves extract, (**b**) Synthesized ZnF, ZnF-CA, and ZnF-CA-LP NCs.

Figure 1a shows UV–visible spectra of an aqueous solution of the crude extract. The extract shows a broad absorption peak at the wavelength of 279 nm, which is assigned to the characteristic absorption of the phenolic groups^66^. Figure 1b displayed the distinct continuous peak absorption pattern of the synthesized magnetite nanoparticles (ZnF, ZnF-CA, ZnF-CA-LP) within the visible spectrum, specifically between 300 and 800 nm. From the absorption spectra pure zinc ferrite nanoparticles are observed in the UV–visible region of 200–500 nm ^67^. Our results show that the peak of UV–vis spectrum of ZnF NPs is at 350 nm. After the lipase is immobilized on ZnF-CA NPs CA using carbodiimide activation of the citric acid carboxylic acid groups, the peak of UV–vis spectrum of ZnF-CA-LP NCs is shifted to 260 nm.

#### Fourier transform infrared spectroscopy analysis

**Figure 2:**
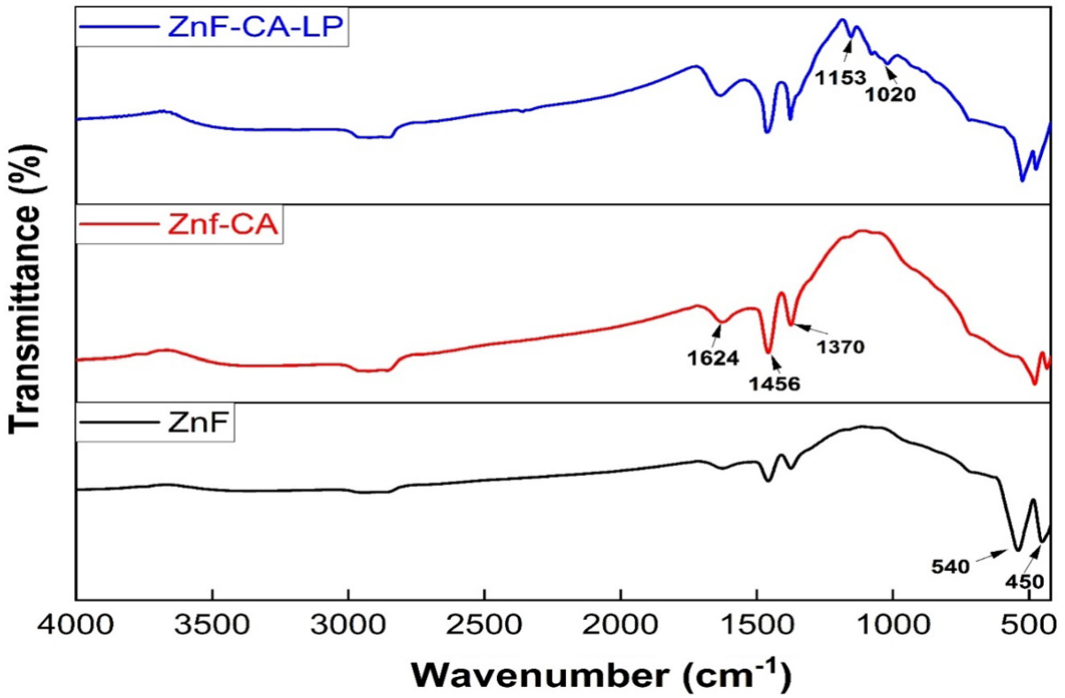
FTIR for the synthesized ZnF NPs, ZnF-CA NPs and ZnF-CA-LP NCs.

Figure 2 illustrates the FTIR absorption spectra of the synthesized ferrite samples. The absorption peak observed at **540** cm^-1^ and **450** cm^-1^ in ZnF is due to the Fe–O and Zn–O vibration in the normal sample respectively. The presence of a broad peak at **3450** cm^-1^ can be attributed to the existence of structural hydroxyl (OH) groups, as well as the presence of molecular water traces. Moreover, the prominent peak observed at **1370** cm^-1^ can be attributed to the C-H vibration band, as well as the peaks observed at **1456** and **1624** cm^-1^, which is attributed to the symmetric stretching of the C = O bond in the COOH group of citric acid, this indicates the binding of a citric acid radical to the surface of ZnF NPs. The presence of bending vibration peaks at **1153 and 1020** cm^-1^ in the CO=N–H amide I region indicates that lipase is immobilized on ZnF-CA (referred to as ZnF-CA-LP)^67,68^ (Fig. 2).

#### X-ray diffraction analysis

**Figure 3:**
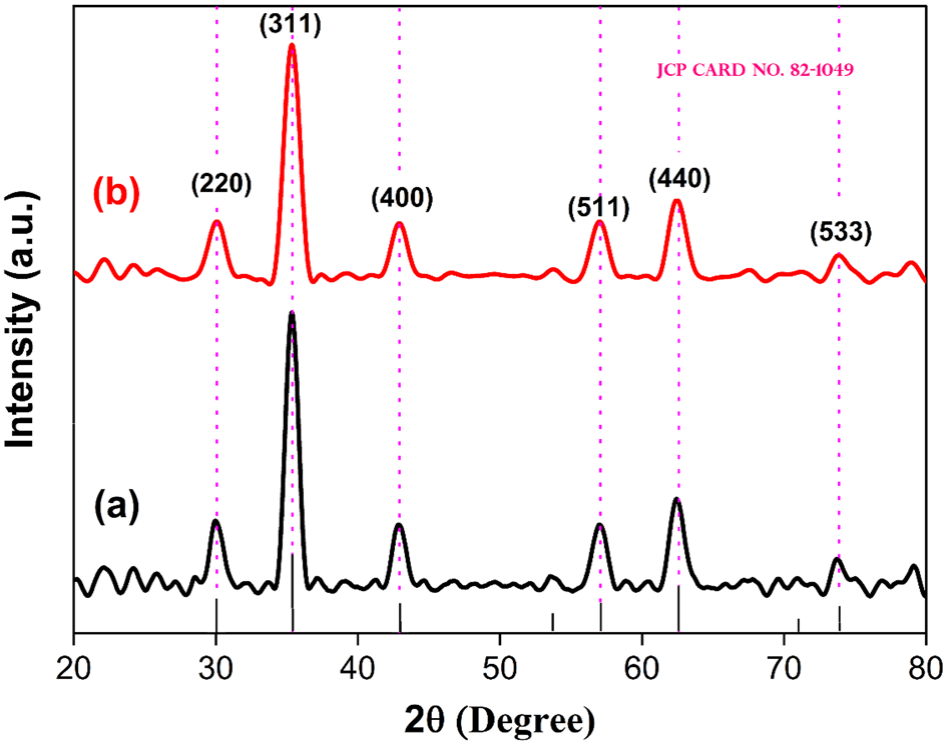
XRD patterns for the synthesized (**a**) ZnF NPs and (**b**) ZnF-CA-LP NCs.

The X-ray diffraction pattern of ZnF NPs and ZnF-CA-LP NCs, as obtained, are depicted in Fig. 3. The current work reports the presence of identical intensity peaks at specific angles, namely 30.12°, 35.54°, 43.13°, 56.89°, 62.62°, and 73.75°. These angles correspond to the reflection planes denoted as (220), (311), (400), (511), (440), and (533) in accordance with references. The work also presents experimental evidence of the behavior exhibited by a cubic structure in the absence of contaminants or significant oxidation. The observed diffraction peaks are well-matched with the standard diffraction data (JCPDS No. 82–1049)^69^ as inset of Fig. 3. The modest deviation from the standard could perhaps be attributed to the distribution of particle sizes and the presence of surface capping. The average crystallite size was deduced by the broadening diffraction peak and calculated using Scherrer’s equation^70^, as listed below.

4 $$D = \, 0.9 \, \lambda / \, \beta \, Cos\theta$$

where, D is the crystallite size, λ is the X-ray wavelength used, β is the full width at half maximum (FWHM) and θ is the diffraction angle. The ZnF-CA-LP NCs crystallite size was 35.0 nm at the (311) plane, which was the strongest peak. The performance of nano photocatalytic substances is greatly impacted by particle size.

#### Scanning electron microscopy analysis

**Figure 4:**
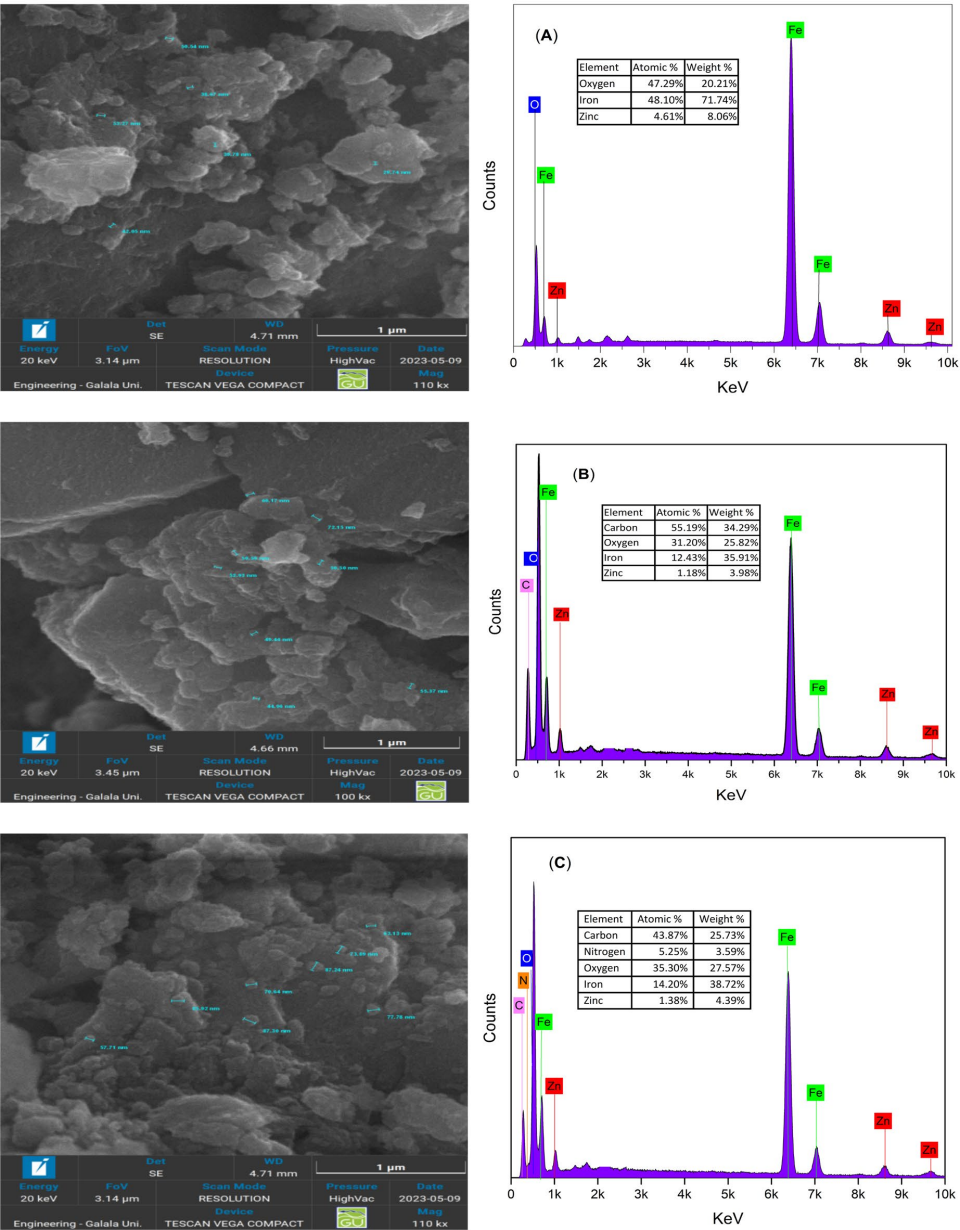
SEM –EDX images of (**A**) Nacked ZnF NPs, (**B**) ZnF-CA NPs, and (**C**) ZnF-CA-LP NCs.

The morphology of the magnetic nanoparticles was examined using TESCAN VEGA COMPACT SEM. Figure 4 shows the uniform distribution and characteristic morphology of the synthesized magnetic ZnF NPs, ZnF-CA NPs & ZnF-CA-LP NCs. The synthesis of ZnF NPs was confirmed by analyzing energy-dispersive X-ray spectroscopy EDX test data, which indicated the presence of Fe, Zn, O. The EDX analysis of ZnF-CA NPs revealed the presence of characteristic EDX peaks corresponding to the atoms of Fe, O, Zn, and C which confirm the coating of citric acid. While the EDX spectra and particular SEM of ZnF-CA-LP NCs reveal the presence of characteristic EDX peaks corresponding to the atoms of Fe, O, Zn, C, and N as a confirmation of amide group formation as documented in previous studies^67,71,72^.

#### Transmission electron microscopy analysis

**Figure 5:**
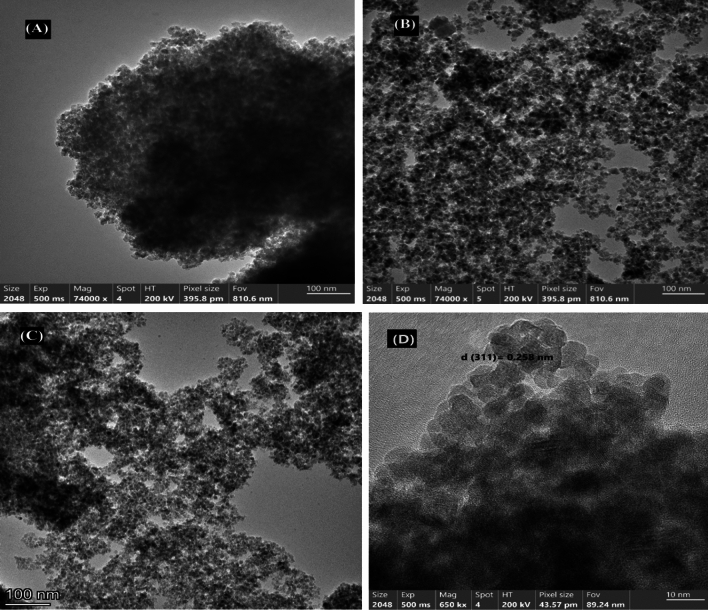
HR-TEM images of (**A**) Nacked ZnF NPs, (**B**) ZnF-CA NPs, (**C**) ZnF-CA-LP NCs and (**D**) HRTEM micrograph showing [311] crystalline lattice measured at the edge of the ZnF-CA-LP NCs crystal.

The synthesized Naked ZnF NPs exhibit a semi-spherical morphology and are characterized by tiny dimensions, as depicted in Fig. 5A. The measured sizes ranged from 20.5 to 45.0 nm, with an average particle size of 25.0 nm. In contrast, ZnF-CA NPs typically exhibit a spherical morphology characterized by smooth surfaces and a narrow range of sizes from 25.0 to 50.0 with an average diameter of 32.5 nm, Fig. 5B. Figure 5C shows the typical HRTEM images of ZnF-CA-LP NCs. Figure 5D denotes the [311] crystalline facet of the crystal with an interplanar distance of 0.258 nm. The HRTEM images reveal that the particles exhibit a spherical morphology and possess a consistent size distribution. The size of these particles can be adjusted within the range of 30.5–55.5 nm, with an average diameter of 38.50 nm which is larger than the synthesized nacked ZnF nanoparticles due to the loading of CA and lipase on their surface.

#### Magnetic measurements

**Figure 6:**
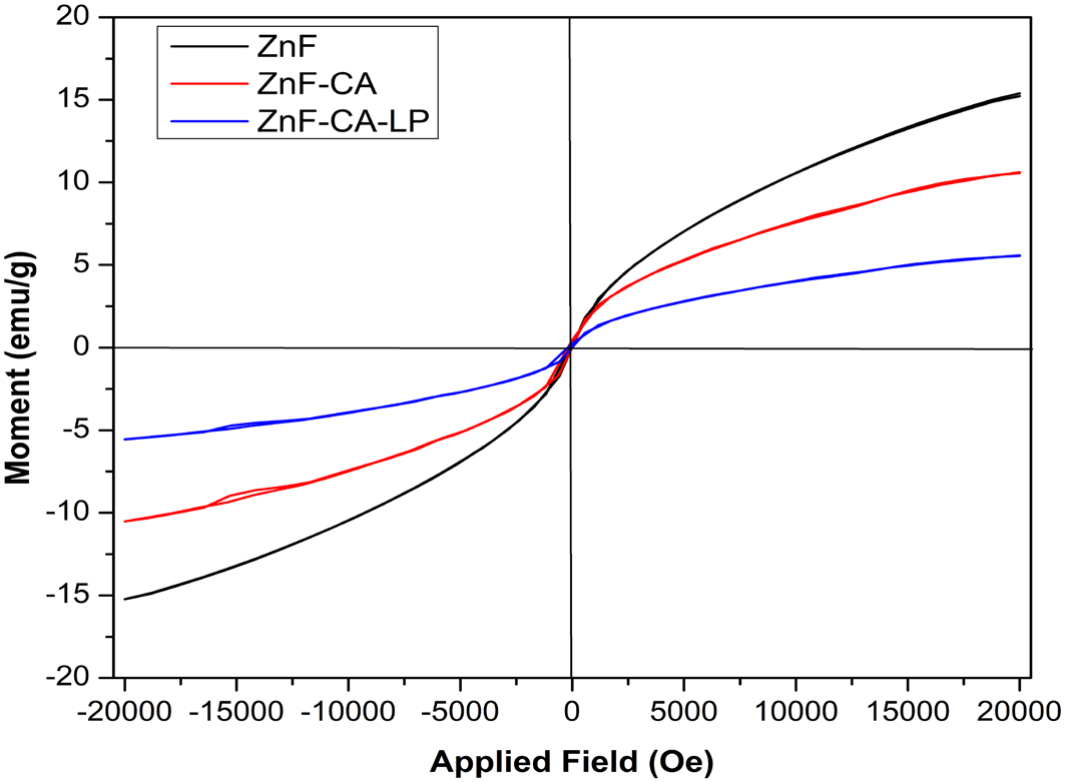
Vibrating sample magnetization of ZnF NPs, ZnF-CA NPs, ZnF-CA-LP NCs.

The magnetic performance of the prepared nanoparticles was also studied with the help of the Vibrating sample magnetometer (VSM), The superparamagnetic properties of the nanoparticles have been confirmed through the absence of the hysteresis loop^73^, as shown in Fig. 6. The Magnetization (Ms) and Remanence (Mr) of ZnF nanoparticles (NPs) are higher compared to that of ZnF-CA NPs and ZnF-CA-LP measuring approximately (15.3, 0.11), (10.3,0.094) and (5.56,0.027) emu/g respectively. While the coercivity (Hc)values for ZnF (37.6 Oe,), ZnF-CA (88.9 Oe), and ZnF-CA-LP (111.08 Oe) are also reported, Table 2.

**Table 2:** Saturation magnetization, Coercivity, and Remanence Magnetization of synthesized NPs.

	Ms (emu/g)	Hc (Oe)	Mr (emu/g)
ZnF	15.3	37.634	0.111
ZnF-CA	10.3	88.95	0.094
ZnF-CA-LP	5.56	111.08	0.027

### Antioxidant activity

**Figure 7:**
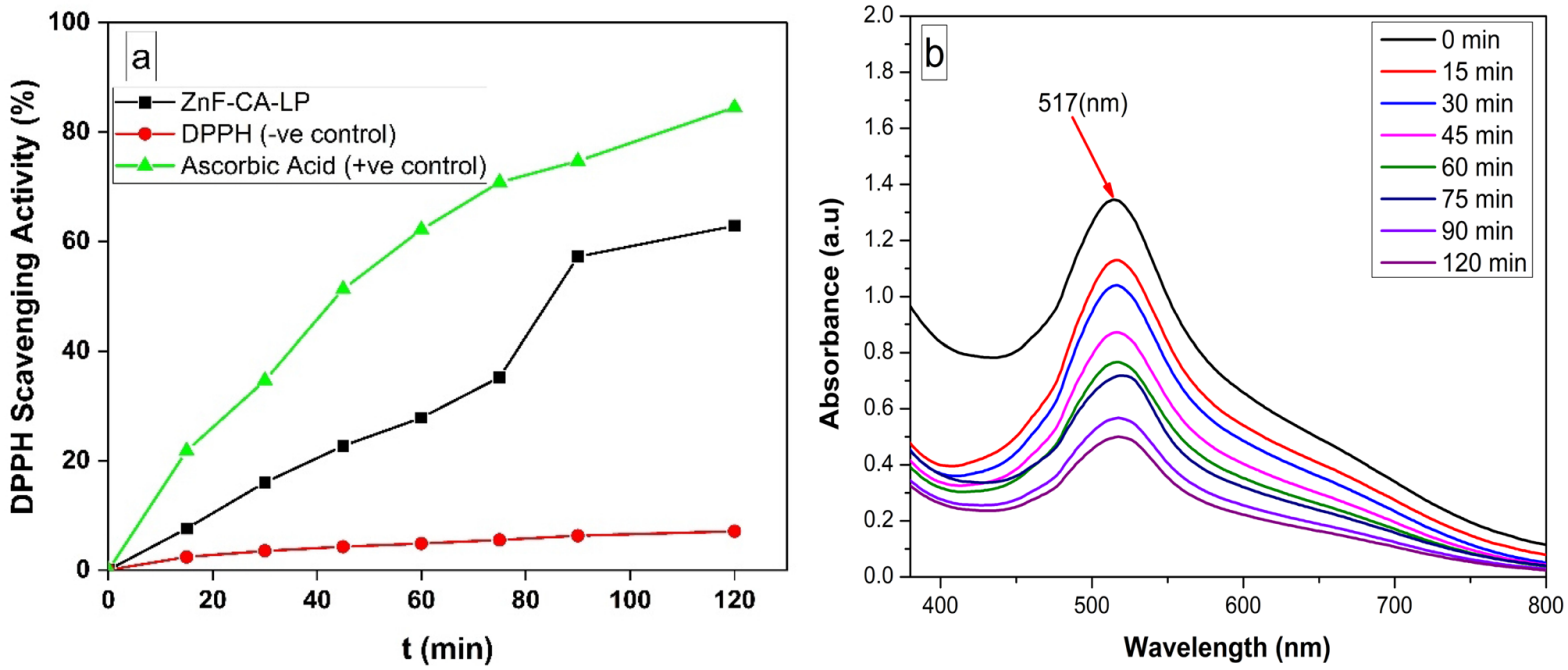
(**a**) DPPH scavenging activity of ZnF-CA-LP NCs, and (**b**) UV–Vis spectrum of DPPH at different concentrations with time intervals.

It is noticed that in the presence of ZnF-CA-LP NCs the color of DPPH solution gradually changes from deep violet to pale yellow. Figure 7b displays the UV–visible spectrum of DPPH at various time intervals in the presence of ZnF-CA-LP NCs. The DPPH scavenging activity of these nanoparticles is determined by measuring the reduction in absorbance at 517 nm^74^. The intensity at 517 nm decreases progressively over time, providing proof of the ZnF-CA-LP NCs' ability to scavenge free radicals. This is illustrated in Fig. 7a. The experiment yielded a result indicating that the ZnF-CA-LP NCs exhibited a DPPH scavenging activity of 62.8% compared to ascorbic acid (positive control) activity of 84.4 within 120 min**.** ZnF-CA-LP NCs possess the ability to transfer their electron density to the free radical situated at the nitrogen atom in DPPH.

### Photocatalytic activity

#### Screening of photocatalytic performance of prepared nanocatalysts

**Figure 8:**
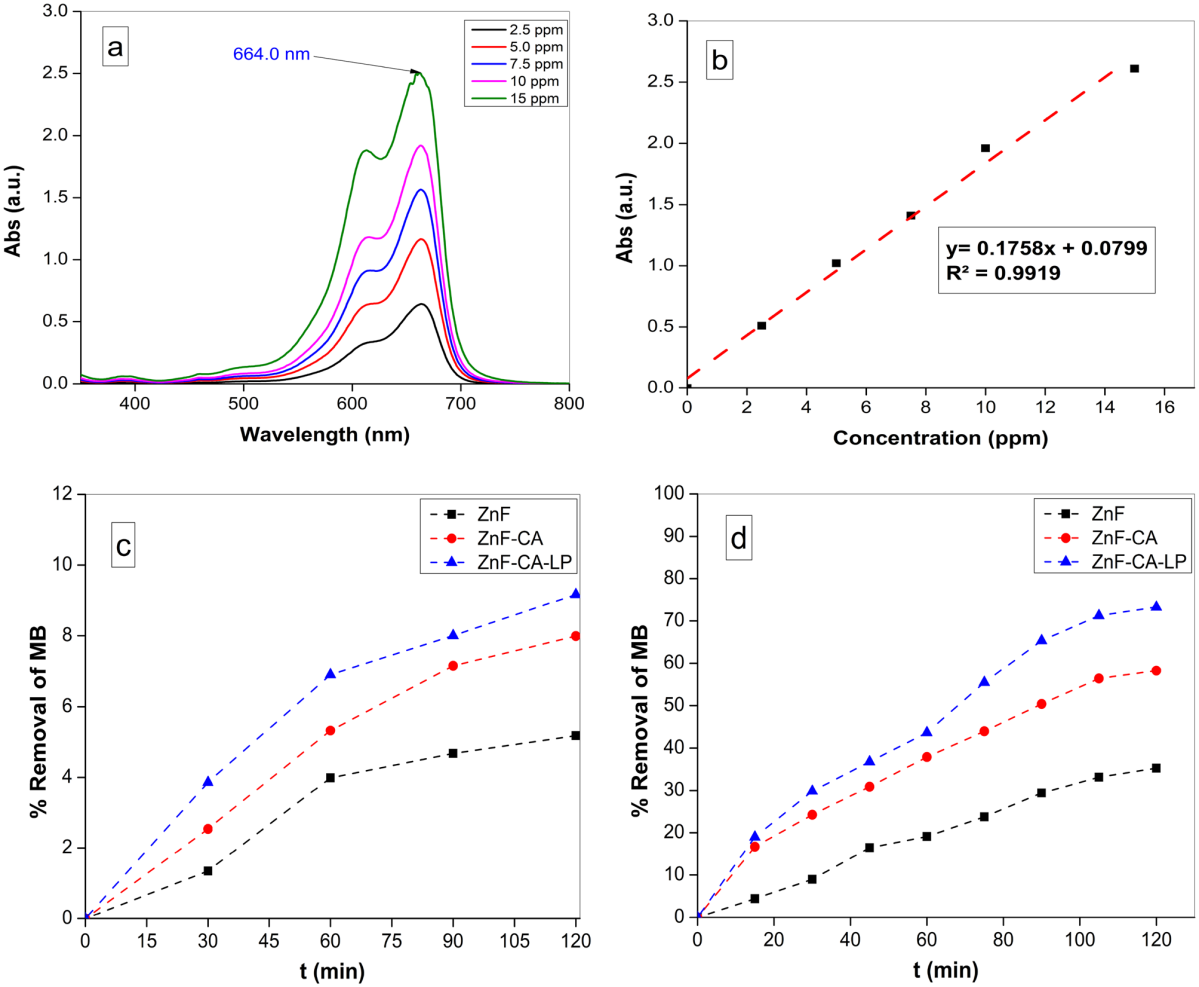
(**a**) UV–Vis spectrum of MB at different concentrations, (**b**) Calibration curve of different concentrations of MB, (**c**) The removal due to adsorption in the absence of light, and (**d**) Removal % of MB under UV photocatalysis using (10 mg nanocatalyst, 50 ml MB solution (10 ppm), Temp. = 25 °C and pH 7.0).

The photocatalytic efficacy of the synthesized samples was determined by assessing their ability to degrade an aqueous solution of Methylene Blue dye (MB, C_16_H_18_ClN_3_S)^75^ under the influence of UV irradiation. The maximum absorption band of Methylene Blue (MB), which was determined at a wavelength of 664 nm^76^, exhibited a steady reduction in intensity over time and upon exposure to ultraviolet (UV) radiation, Fig. 8a. Figure 8b represents the calibration curve of MB obtained by different concentration ranged from 2.5 to 15 ppm. Upon conducting a comprehensive analysis of all the produced magnetic nanoparticles (MNPs), it was determined that ZnF-CA-LP NCs exhibited the highest level of efficacy as a catalyst for the photodegradation of MB after 120 min as shown in Fig. 8d. About 35.2, 58.4 and 73.5% of the MB had been eliminated after subjecting it to 120 min of ultraviolet (UV) irradiation using ZnF NPs, ZnF-CA NPs, and ZnF-CA-LP NCs; respectively. The removal due to adsorption in the absence of light during the same timeframe was approximately 5.1, 7.9, and 9.2% using ZnF, ZnF-CA and ZnF-CA-LP NCs, respectively, as shown in Fig. 8c.

#### Effect of initial pH

**Figure 9:**
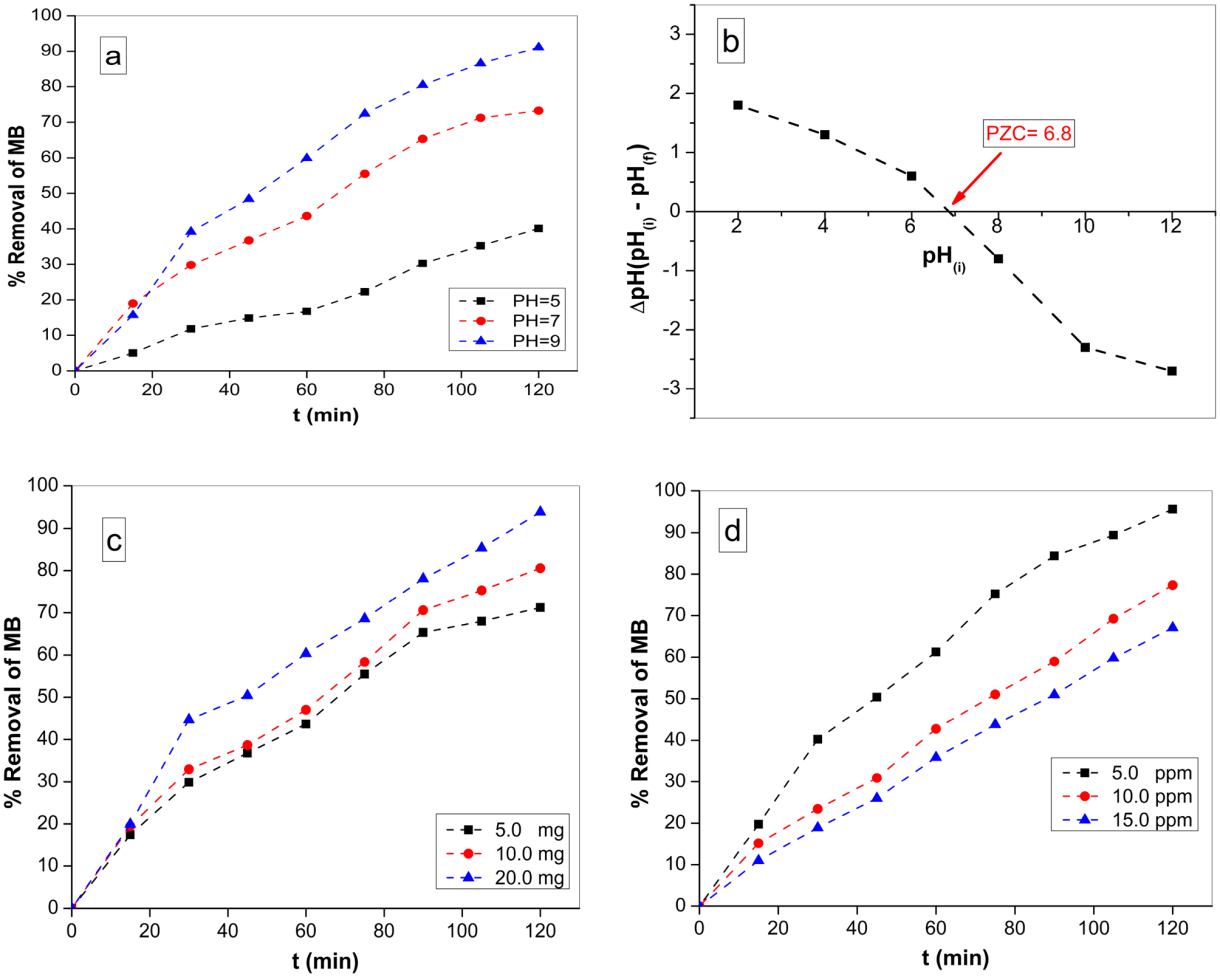
(**a**) Effect of different initial pH values, (**b**) Point of Zero charge (PZC), (**c**) Effect of the photocatalyst dose on the removal efficiency of MB (50 ml MB solution (10 ppm), temp. = 25 ^°^C and pH 7.0), and (**d**) Effect of different MB concentration (5, 10 and 15 ppm) at pH 9.0 and 10.0 mg ZnF-CA-LP NCs.

This section focuses on the investigation of the impact of pH on the process of photocatalytic degradation. The pH values in this study range from 5.0 to 9.0, and the experiments were conducted at the ambient temperature (25 °C). Fifty ml of MB solution with a concentration of 10.0 ppm was subjected to stirring. Figure 9a illustrates the influence of the initial pH values on the photodegradation of MB under irradiation for 120 min. The findings indicated that the removal of MB was enhanced at higher pH values^77^. The degradation of MB is a multistage process, with the initial phase including the adsorption of MB onto the catalyst. The surface electrical properties of magnetic nanoparticles (MNPs) were found to be responsible for their photocatalytic potential, which was influenced by the presence of several interlayer anions^78^. The enhanced surface potential of ZnF-CA-LP NCs facilitated the adsorption of MB, hence enhancing the efficiency of charge generation induced by light. The initial pH level has the potential to alter both the surface charge and agglomeration of photocatalysts.

#### Point of zero charge

The determination of the point of zero charge (PZC) involved the addition of 10 mg of ZnF-CA-LP NCs to a 50 mL solution of 0.01 M NaCl. The pH values of the solutions were adjusted to 2, 4, 6, 8, 10, and 12 with the addition of HCl or NaOH. The samples were subjected to agitation at a speed of 200 rpm for 48 h. After the ZnF-CA-LP NCs were subjected to magnetic separation, the pH values of the solutions were determined. The pH at the PZC was determined by utilizing a graph that correlates the initial pH with the end pH^79^. According to Fig. 9b, it can be observed that the pH at the PZC was identified as 6.8, as there was no substantial disparity between the final and initial pH readings. This observation indicates that the surface charge of the photocatalyst is positive when the pH is lower than the point of zero charge (PZC), and negative when the pH is higher than the PZC. Moreover, when the pH of the solution matches the pH at the point of zero charge (PZC), the surface charge of the photocatalyst becomes neutral, leading to a minimal electrostatic attraction between the photocatalyst surface and ions^80^.

The observed outcome elucidated the reason behind the heightened efficiency of the photocatalytic degradation of MB at a pH of 9.0, as depicted in Fig. 9a. The ZnF-CA-LP NCs have a negative net surface charge, hence facilitating the attraction of positively charged MB molecules. This electrostatic interaction accelerates MB degradation by photocatalysis. The rate of photocatalytic degradation of MB exhibited a deceleration as the pH value reached 5. The positive net surface charge of the ZnF-CA-LP NCs at pH = 5 results in repulsion between the positive charge of MB and the positive surface charge of the nanocomposite.

#### Effect of the photocatalyst dose

This study examines the impact of ZnF-CA-LP NCs concentration on the efficiency of MB degradation following exposure to UV radiation. The dosage of the catalyst varied between 5.0, 10.0 and 20.0 mg. The results of this study indicate that there was an increase in the removal rate when the dose of photocatalyst was increased at 120 min with a range of 71.2% to 93.8% Fig. 9c. It is believed that a higher concentration of photocatalyst in the reaction results in a larger number of active sites on the photocatalyst compared to the volume of the MB solution^81^.

#### Effect of dye concentration

Figure 9d shows the effect of different concentrations (5.0, 10.0 and 15.0 ppm) of MB dye on the dye degradation as indicated by decolorization, with fixation of other parameters in the reaction. The results demonstrate that decolorization was more effective at a lower dye concentration (5.0 ppm), albeit a substantial decline in decolorization occurring at a greater concentration (15.0 ppm) of dye. This matches with the results of other researchers^82,83^. The suggested explanation is that when the concentration of the dye increases, with a constant concentration of hydroxyl radicals; a lower rate of elimination takes place^84^. The dye molecules must undergo adsorption onto the catalyst prior to interacting with the active iron ion sites on the catalyst. An elevation in dye concentration led to a competition for active sites, resulting in a minor reduction in the percentage of color removal^85^. Furthermore, a high concentration of dye also diminishes the ability of light to penetrate the dye solution, resulting in a decrease in the generation of OH radicals^86^.

#### ***Effect of H***_***2***_***O***_***2***_

**Figure 10:**
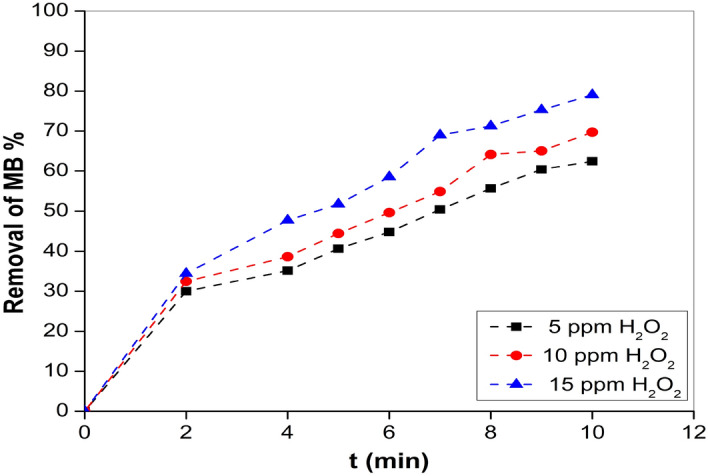
Effect of different concentrations of H_2_O_2_ and different time points.

In order to investigate the impact of hydrogen peroxide concentrations on the rate of decolorization of MB dye through UV photolysis, a range of H_2_O_2_ concentrations were tested to determine its enhancing effect^87^. The process of generating ^•^OH can be accelerated by augmenting the quantity of H_2_O_2_ in the initial mixture, as H_2_O_2_ serves as a ^•^OH source upon exposure to UV radiation^88^. The present study conducted a thorough investigation on the efficacy of UV/H_2_O_2_ in the removal of MB dye. Various concentrations of H_2_O_2_ (5, 10 and 15 ppm) were employed, along with a 10 mg sample of ZnF-CA-LP NCs, and a consistent concentration of MB dye (10 ppm at pH 9). The duration of the treatment was set at 10 min. The objective was to identify the optimal circumstances for effectively treating varied quantities of MB dye. The augmentation of H_2_O_2_ quantities significantly facilitated the removal of the MB dye. The H_2_O_2_ concentrations at 5, 10, and 15 ppm were associated with a corresponding increase in the % removal of MB dye (62.47%, 69.7%, and 79.07% respectively. The removal efficiency of the MB dye increased proportionally with the increase of H_2_O_2_ concentration with the increasing of time as depicted in Fig. 10.

#### Mechanism of photocatalysis of MB

**Figure 11:**
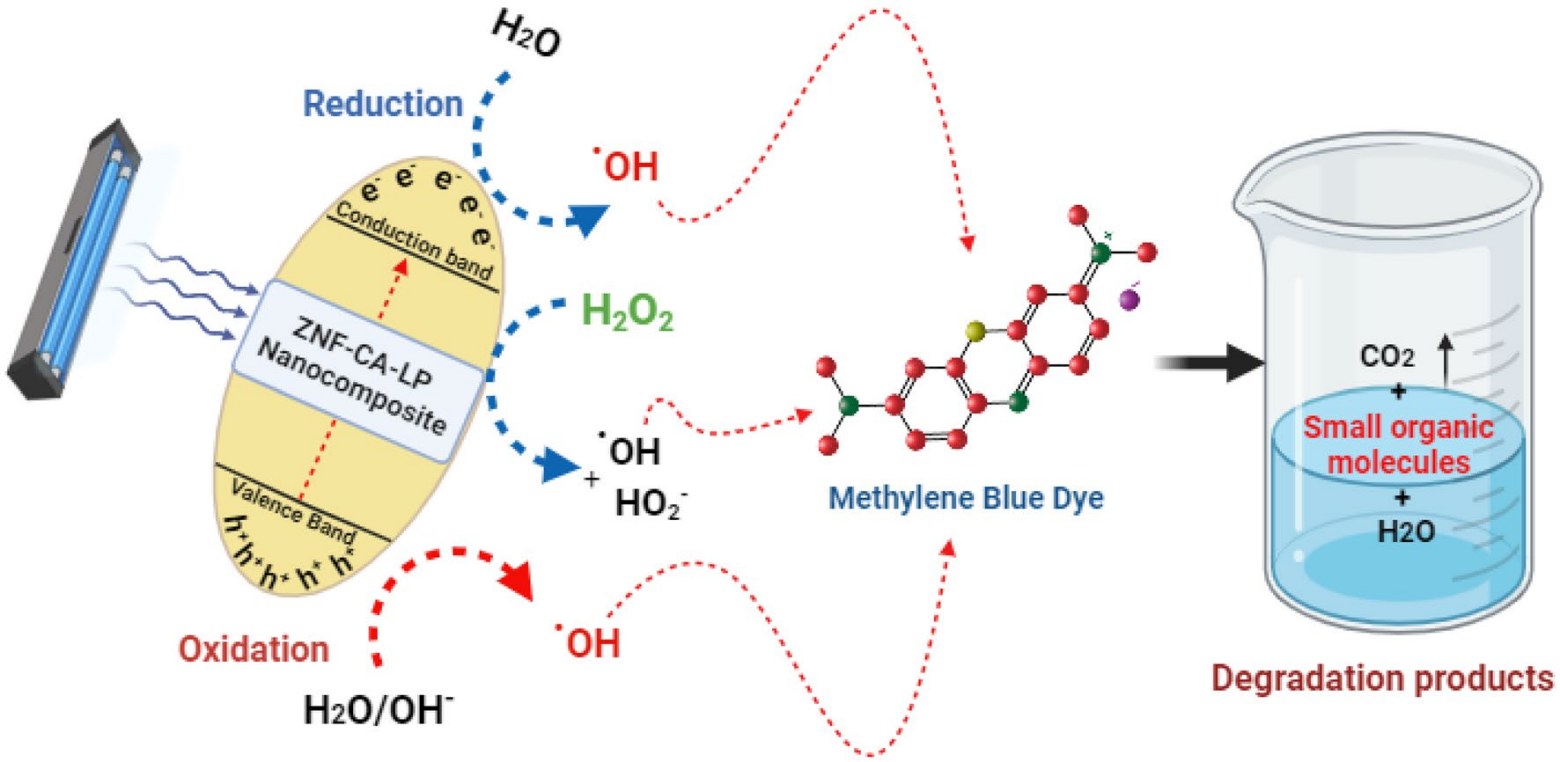
Proposed mechanism for the photocatalytic degradation of MB by the synthesized ZnF-CA-LP NCs in the presence of H_2_O_2_.

As mentioned in several studies of works of literature^89–91^, the possible mechanism is as follows; When photons, in the form of light rays, interact with a material and have energy that is equal to or greater than its bandgap, electrons in the conduction band (CB) move to the valence band (VB) by crossing the bandgap, resulting in the creation of positive holes, Consequently, this results in the production of reactive oxygen species (ROS), which is the most important consequence of photocatalysis because it has an effect on the environment and is therefore utilized in the degradation of MB dye. The active hydroxyl radicals (^**.**^OH) function as powerful oxidizing agents, efficiently breaking down MB molecules to produce the ultimate oxidation products. It is important to highlight that the combination of ZnF-CA-LP NCs with H_2_O_2_ has a synergistic effect, leading to the generation of^**.**^OH and HO_2_^**.**^ radicals. The possible photocatalytic mechanism is described in Fig. 11.

#### Reusability of the ZnF-CA-LP NPs

**Figure 12:**
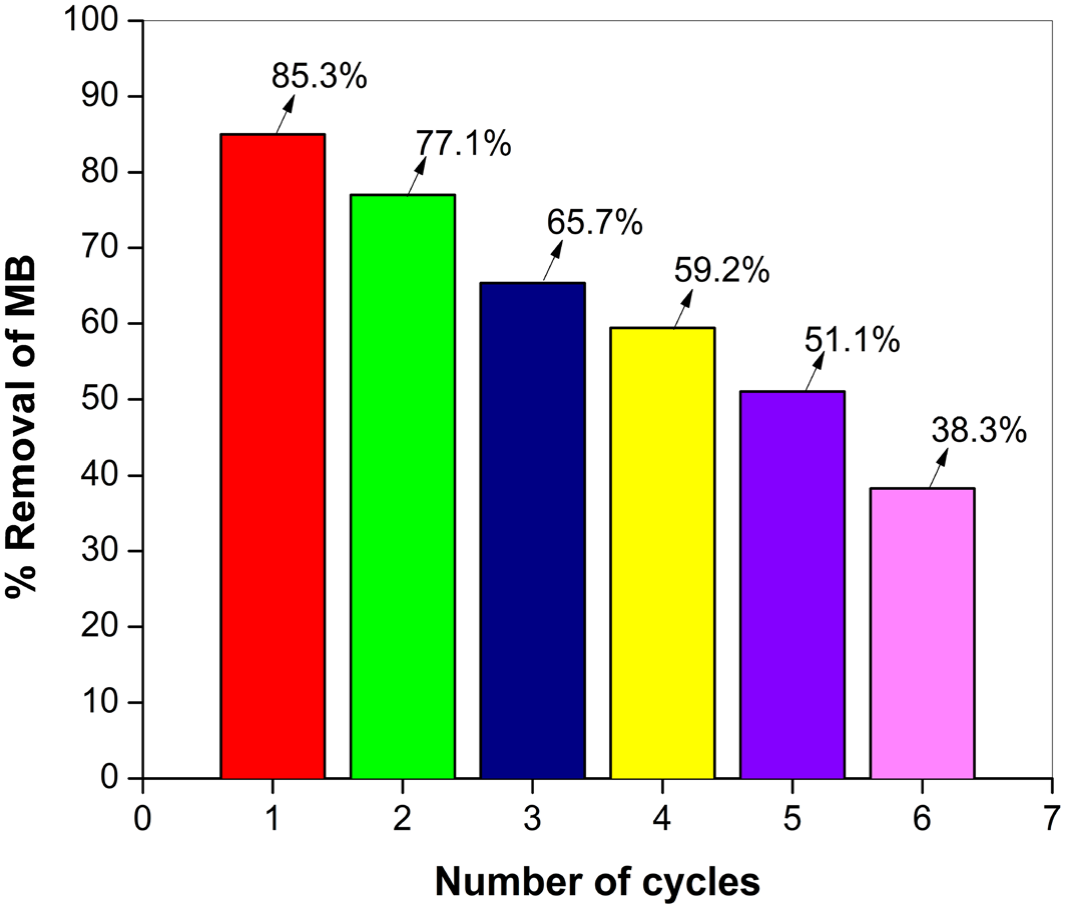
Recyclability of ZnF-CA-LP NCs for MB degradation under UV irradiation.

For practical applicability, the reusability of immobilized enzyme is considered an essential component that plays a significant role in making a method economically viable and possible^7^. This suggests that suitable reusability of ZnF-CA-LP NCs in successive cycles of application is an important aspect to study. Therefore, a supplementary investigation was undertaken to examine the potential reusability of ZnF-CA-LP NCs in the context of photocatalytic reduction of MB dye when subjected to UV irradiation. The photocatalytic reuse experiments were conducted using the identical settings as those stated in the earlier evaluation of photocatalytic activity. The dye degradation rates in the initial and subsequent 5 cycles were found to be 85.3%, 77.1%, 65.7%, 59.2%, 51.2%, and 38.3%, respectively as shown in Fig. 12. Before its utilization in the subsequent cycle, ZnF-CA-LP NCs were subjected to centrifugation, followed by washing with deionized water and thereafter allowed to undergo overnight drying. The decrease in residual activity seen over six cycles may be attributed to the agglomeration of nanomaterials, the leaking of enzymes from the supporting materials, and the inactivation of the enzyme^92^.

### Kinetic studies

**Figure 13:**
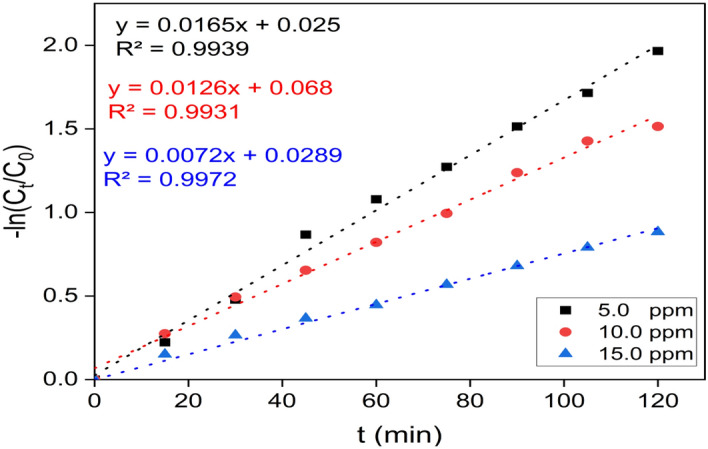
A linear fit, first-order model data is reported in kinetic form for MB degradation under UV irradiation with beginning MB concentrations of 5, 10, and 15 ppm.

The photocatalytic activity of a catalyst is contingent upon its intrinsic structural characteristics^93^. The degradation kinetics of MB were investigated by utilizing photocatalysts consisting of ZnF-CA-LP NCs under the influence of UV radiation. The rate of MB degradation can be determined using the following Eq. (5)

5 $$- {\text{ln }}\left( {{\text{C}}_{{\text{t}}} /{\text{ C}}_{0} } \right) \, = - {\text{Kt}}$$

where (C_t_ and C_0_) are the respective initial and residual concentrations of MB, t is the removal interval, and k is the removal rate constant. Figure 13. shows the relationship between (− ln C_t_/C_o_) Vs. t (min). The findings showed that pseudo-first-order rate laws were obeyed by the removal process's kinetics. Moreover, the apparent pseudo-first-order rate constants drop with increasing catalyst dosage.

### Antibacterial and antibiofilm activities of the synthesized nanocomposites

#### Antibacterial activity

**Figure 14:**
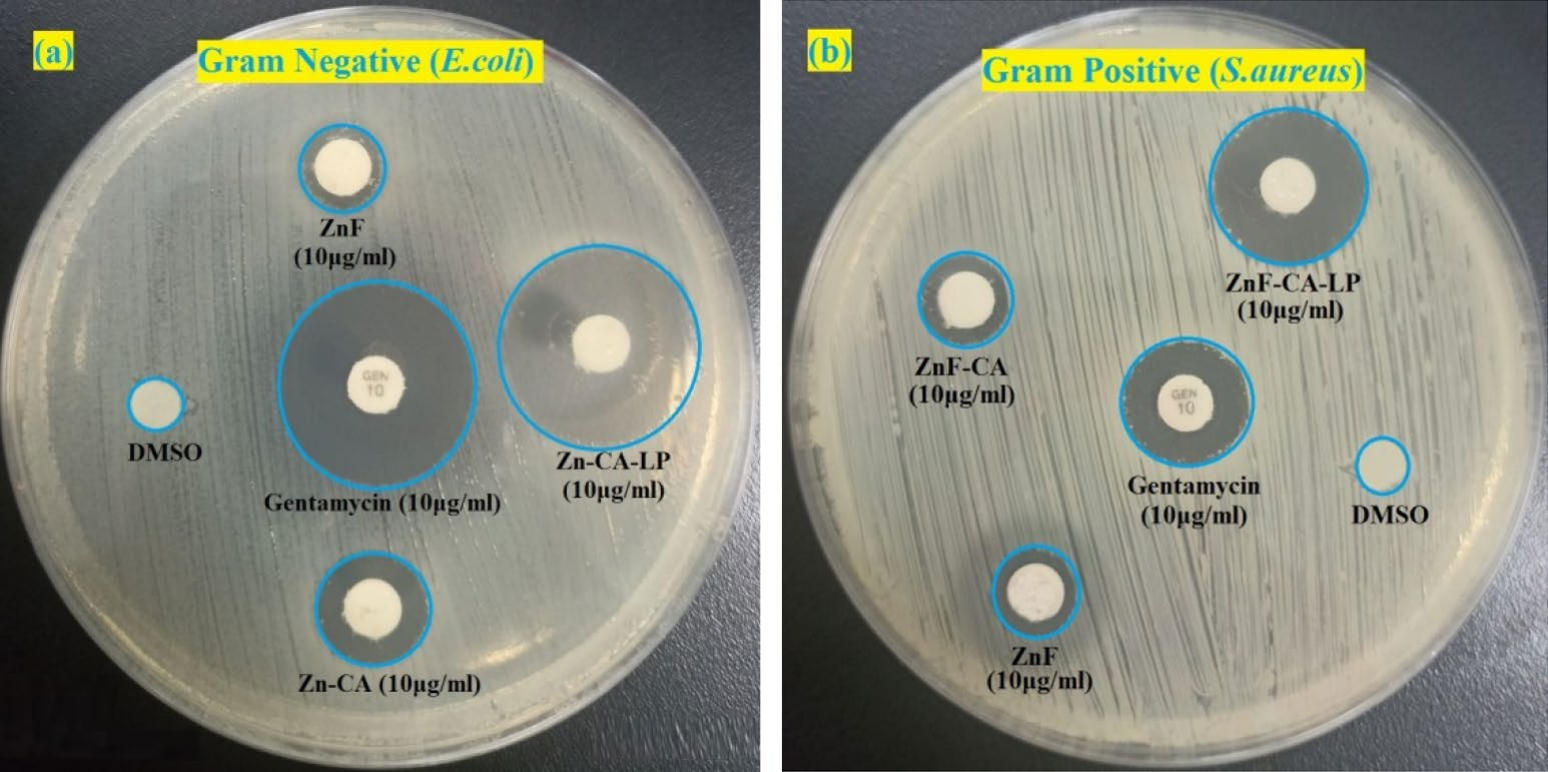
Antimicrobial activity as ZOI for ZnF NPs, ZnF-CA NPs, and ZnF-CA-LP NCs against (**a**) Gram-negative (*E. coli*), and (**b**) Gram-positive (*S. aureus*) bacteria, using standard antibiotic Gentamycin (GEN) as positive control and DMSO as negative control.

The antibacterial efficacy of the nanoparticles that were synthesized was evaluated by employing the agar well diffusion technique against a single strain of gram-positive bacteria *(S. aureus*) as well as a single strain of gram-negative bacteria *(E. coli).* The assessment of ZOI was performed for each bacterial strain about the positive control (GEN), and the negative control, (DMSO). The lack of microbial proliferation in the proximity of NPs can be interpreted as an indirect indication of the NPs' capacity to hinder or suppress microbial growth^94^. The disc agar distribution method, employed as a screening experiment, has revealed that the ZnF-CA-LP nanocomposite has a qualitative antibacterial efficacy against the bacteria tested, as depicted in Fig. 14. The results of the in-vitro ZOI demonstrate that ZnF-CA-LP NCs exhibit significant antibacterial activity against *S. aureus* and *E. coli* with a ZOI of 16.0 and 20.0 mm, respectively as shown in Table 3. It is observed that the antibacterial efficacy of the ZnF-CA-LP NCs is considerably more than that of ZnF-CA and ZnF NPs, indicating the possible synergistic effect between Lipase and ZnF-CA NPs. The antibacterial capabilities of nanocomposites cannot be only determined by their size; other significant factors such as elemental composition, purity, surface area, and form, require careful consideration^95^. ZnF-CA-LP NCs have several advantages, including a significant surface-to-volume ratio and a nano-scale structure, which facilitate their interaction with biological entities such as bacteria and yeast. The minimum inhibitory concentration (MIC) values of ZnF-CA-LP NCs, ZnF-CA NPs, and ZnF NPs against the different bacteria under study are reported in Table 1 ZnF-CA-LP NCs exhibit significant MIC values of 1.25 μg/mL and 0.625 μg/mL against *S. aureus* and *E. coli,* respectively.

**Table 3:** Antimicrobial activities for ZnF NPs, ZnF-CA NPs, and ZnF-CA-LP NCs, against *S. aureus* and *E. coli* measured as ZOI (mm), MIC (µg/ml) and antibiofilm activity of ZnF-CA-LP NCs.

Bacterial strains	ZOI of ZnF NPs (10.0 µg/ml) (mm)	ZOI of ZnF-CA NPs (10.0 µg/ml) (mm)	ZOI of ZnF-CA-LP (10.0 µg/ml) (mm)	ZOI of gentamycin (10.0 µg/disc)mm	MIC of ZnF-CA-LP NPs (µg/ml)	Antibiofilm (%)
*S. aureus*	9.0 ± 0.477	11.0 ± 0.351	20.0 ± 0.512	16.0 ± 0.323	1.250	88.4
*E. coli*	8.0 ± 0.286	13.0 ± 0.425	27.0 ± 0.651	26.0 ± 0.281	0.625	96.6

#### Anti-biofilm activity

**Figure 15:**
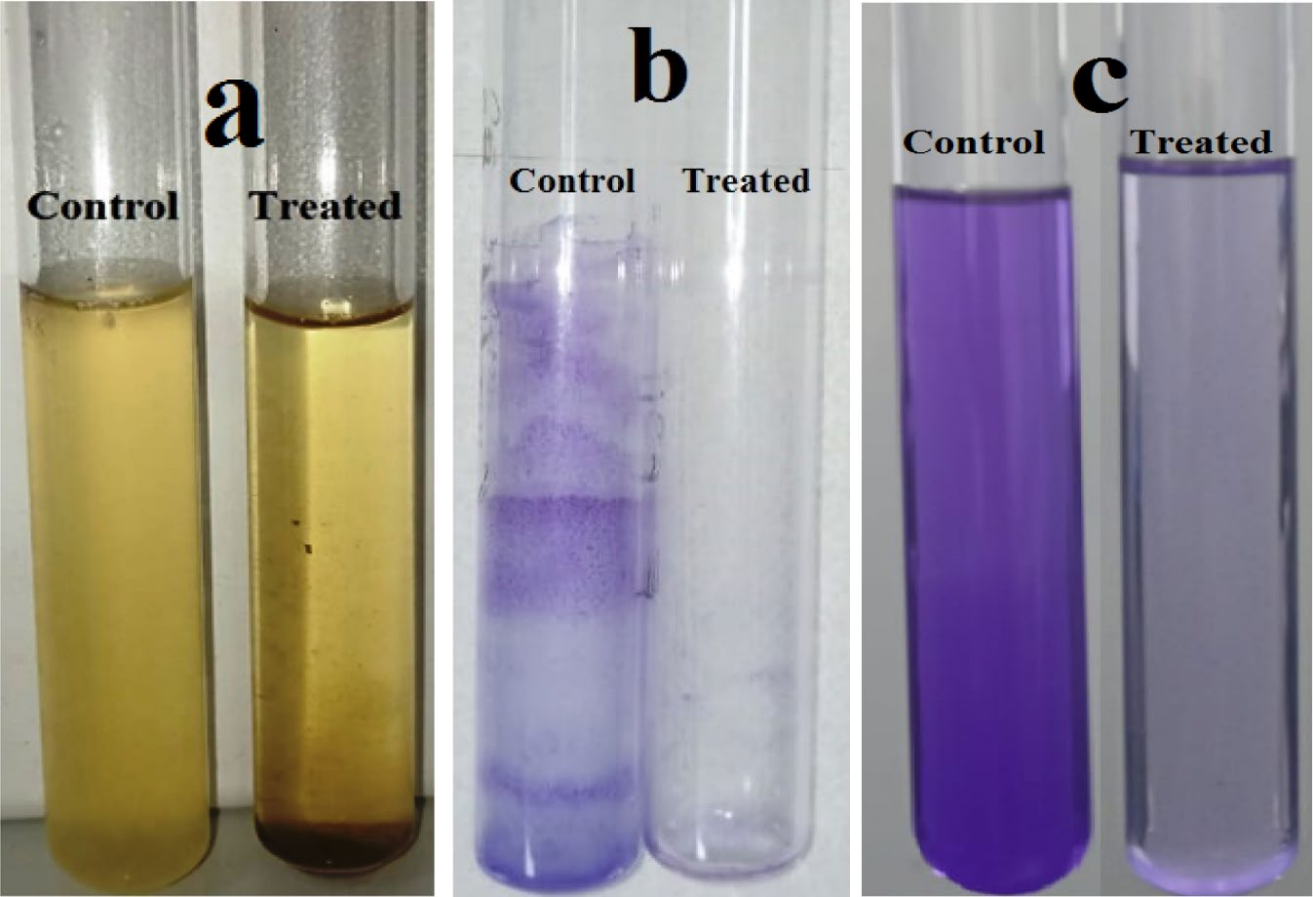
The antibiofilm efficacy of ZnF-CA-LP NCs was evaluated against *E. coli* utilizing the test tube method. The reported sequence of actions is as follows. (**a**) The proliferation of bacterial cells and the development of biofilm (rings) in the absence of ZnF-CA-LP NCs treatment, and the suppression of bacterial growth following treatment with ZnF-CA-LP NCs. (**b**) Application of crystal violet to the bacterial cells that are attached to the surface. (**c**) Ethanol is used to eliminate and dissolve the attached bacterial and yeast cells to determine the semi-quantitative suppression of biofilm formation (%).

The production of biofilms has been observed in many microorganisms that produce exopolysaccharides^96–98^. Figure 15 shows the antibiofilm activity of ZnF-CA-LP NCs against *E. coli* bacteria (as a model for susceptible bacteria) using a test tube method. *E. coli* displayed a concentrated whitish-yellow film that covered the entirety of the interface between air and liquid in the absence of ZnF-CA-LP NCs as shown in Fig. 15a. It demonstrated strong adherence to the inner wall of the test tubes and appeared as a circular blue structure after being stained with crystal Violet (CV), as shown in Fig. 15b. A suspension of blue color was produced by dissolving the ring stained with CV using 99.0% ethanol, Fig. 15c. On the other hand, the test tubes that were inoculated with *E.coli* and subjected to treatment with ZnF-CA-LP NCs with a concentration of 10.0 µg/ml exhibited a significant inhibitory impact. This was evident from the observed limited proliferation of bacterial rings, as depicted in Fig. 15a. The optical density was measured subsequently at a wavelength of 570.0 nm^99^.

Table 3 presents the data on the percentage of inhibition observed in the biofilms generated by the bacteria under investigation. The most significant level of inhibition was seen against *E.coli* (96.6%), followed by *S.aureas* (88.4%), during exposure to a concentration of 10.0 µg/ml of the ZnF-CA-LP NCs.The observed variation in the inhibition percentage can be attributed to various factors such as antimicrobial properties, biosorption capacity, physical characteristics, invasive potential, and distinctive chemical properties that regulate the interaction between the nanomaterials and the biofilms^29^.

#### Mechanism of antimicrobial activity of the synthesized ZnF-CA-LP NCs

Studies have documented the effectiveness of metal nanoparticles as antibacterial agents, and the harmful effects of metals can be evaluated in both bacterial and eukaryotic organisms^100^. Nevertheless, there is a lack of reports on enzyme-metal nanocomposites, and the specific chemical mechanism for their antibacterial action remains unclear^101^. The antibacterial mechanism of metal nanoparticles involves the disruption of the cell membrane, the generation of reactive oxygen species (ROS) that degrade lipids, proteins, or DNA, and the inhibition of bacterial metabolism, ultimately leading to bacterial death. The enzyme-metal nanocomposite exhibits a synergistic effect in dismantling cellular structures and impeding the proliferation of bacteria^102^.

**Figure 16:**
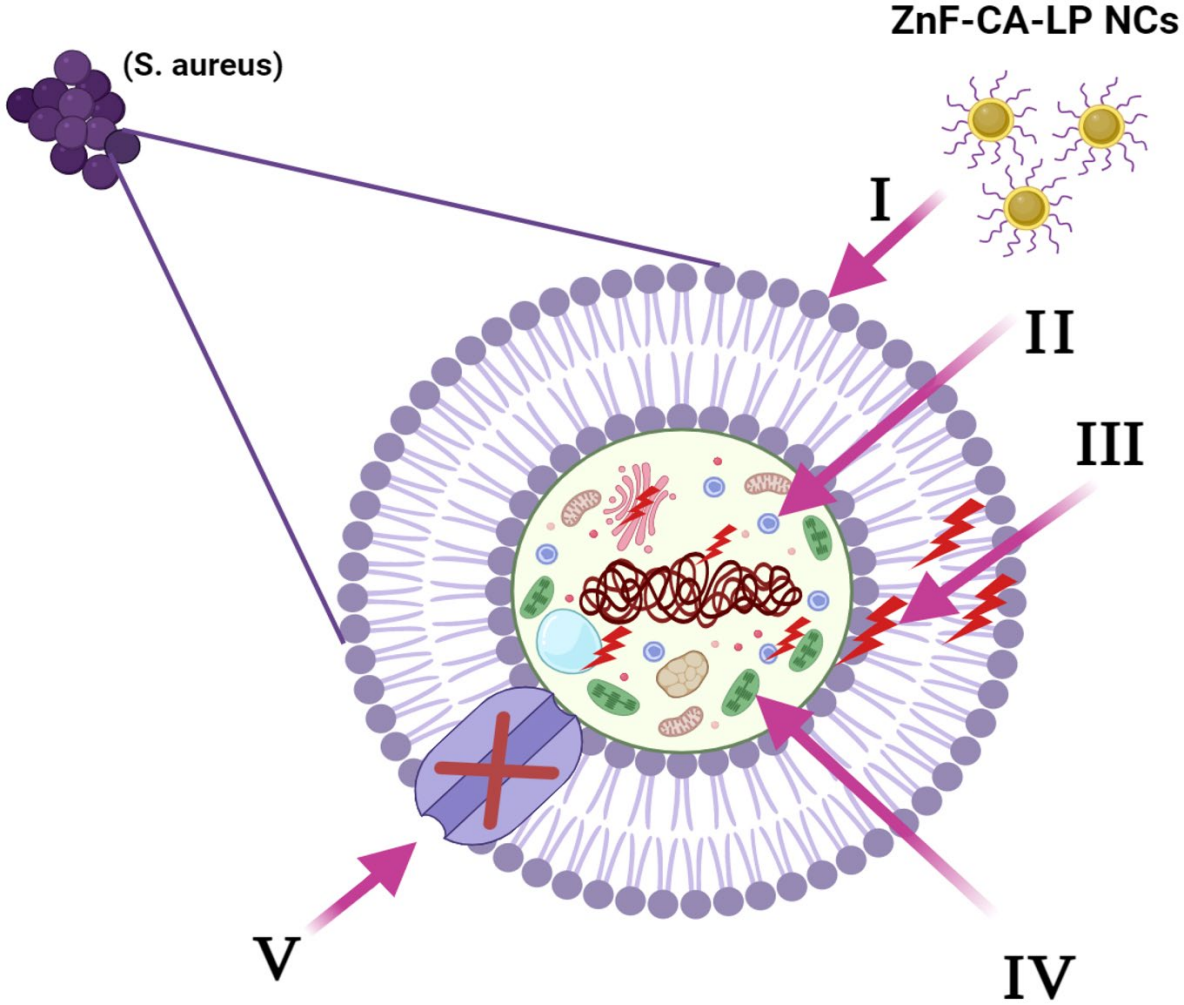
The diagram illustrates the five primary mechanisms by which ZnF-CA-LP NCs exhibit their antibacterial properties. **(I)** The ZnF-CA-LP NCs stick to and attach the surface of the microbial cell, leading to the release of Lipase, which in turn causes damage to the cell membrane and changes in its transport activity. **(II)** ZnF-CA-LP NCs infiltrate the microbial cells and engage with intracellular biomolecules, thereby influencing the corresponding cellular machinery. **(III)** The presence of ZnF-CA-LP NCs induces the generation and amplification of ROS, resulting in cellular damage. **(IV)** The ZnF-CA-LP NCs manipulate the cellular signal pathway and induce cell death. **(V)** ZnF-CA-LP NCs effectively inhibit the movement of ions into and out of microbial cells.

A comprehensive overview was provided for the various processes by which enzyme-metal nanocomposites exert their antibacterial effects, encompassing both extracellular and intracellular pathways, according to the antibacterial properties of nanomaterials. Enzyme-metal nanocomposites, composed of specific hydrolase, can break down extracellular polymeric substrates (EPS) that consist of protein, polysaccharides, and DNA in the external environment. Simultaneously, enzyme-metal nanomaterials can hydrolyze and oxidize peptidoglycan, lipids, and proteins found on the majority of bacterial cell walls and membranes. Enzyme-metal nanocomposites can also produce ROS and disrupt the function of lipids, proteins, and DNA within bacterial cells, hence impacting bacterial metabolism, Fig. 16.

## Conclusion

ZnFe_2_O_4_ NPs have been synthesized by the green synthesis method using *Psidium guava* leaf extract as a reducing agent and characterized by structural and optical tools. The surface of ZnFe_2_O_4_ NPs was coated with citric acid then the lipase enzyme was immobilized to obtain modified ZnF-CA-LP NCs. The photocatalytic efficiency of the synthesized ZnF-CA-LP NCs was tested against Methylene blue (MB). Also, different parameters affecting the efficiency of removal potential such as pH, MB concentration, and photocatalyst dose have been studied. According to XRD, SEM, and TEM analyses, it was found that ZnF NPs were located at the core, while the citric acid and lipase enzyme were coated on this core, producing ZnF-CA-LP NCs with particle sizes varying from 30.5 nm to 55.5 nm with average 38.50 nm which was larger than the synthesized nacked ZnF NPs due to the loading of CA and lipase on their surface. The ZnF-CA-LP NCs were applied for MB removal as an indicator of its applicability in wastewater treatment. The ZnF-CA-LP NCs had the highest efficiency for MB removal, achieving 96.0% removal of 5.0 ppm of MB at pH 9, and had a reusability for six successive cycles. In addition, we investigated the anti-bacterial activity against *S. aureus* and *E. coli*, and demonstrated that ZnF-CA-LP NCs exhibited significant MIC values of 0.312 μg/mL and 0.625 μg/mL against *S. aureus* and *E. coli*, respectively. Furthermore, the nanocomposites had a significant antibiofilm activity against *S. aureus* (88.4%) and *E. coli* (96.6%). Hence, ZnF-CA-LP NCs are considered promising biocatalysts for potential applications in environmental and biomedical uses. various types of green synthesized metal oxide NPs (ZnO, CuO, TiO_2_, and ZnO/CuO) for different applications need to have more attention in the future.

## References

[1] Rónavári A (2021). Green silver and gold nanoparticles: biological synthesis approaches and potentials for biomedical applications. Molecules.

[2] Mukherjee A, Sarkar D, Sasmal S (2021). A review of green synthesis of metal nanoparticles using algae. Front. Microbiol..

[3] Jeevanandam J (2022). Green approaches for the synthesis of metal and metal oxide nanoparticles using microbial and plant extracts. Nanoscale.

[4] Patra JK (2018). Nano based drug delivery systems: recent developments and future prospects. J. Nanobiotechnol..

[5] El-Khawaga AM (2023). Green synthesized ZnO nanoparticles by Saccharomyces cerevisiae and their antibacterial activity and photocatalytic degradation. Biomass Convers Biorefinery.

[6] Fahim YA (2024). A review on Lipases: sources, assays, immobilization techniques on nanomaterials and applications. BioNanoScience.

[7] Chapman J, Ismail AE, Dinu CZ (2018). Industrial applications of enzymes: Recent advances, techniques, and outlooks. Catalysts.

[8] Abdulmalek SA, Yan Y (2022). Recent developments of lipase immobilization technology and application of immobilized lipase mixtures for biodiesel production. Biofuels, Bioproducts Biorefining.

[9] Filho DG, Silva AG, Guidini CZ (2019). Lipases: sources, immobilization methods, and industrial applications. Appl. Microbiol. Biotechnol..

[10] Zhang H, Secundo F, Sun J, Mao X (2022). Advances in enzyme biocatalysis for the preparation of functional lipids. Biotechnol. Adv..

[11] Muteeb G (2023). Origin of antibiotics and antibiotic resistance, and their impacts on drug development: a narrative review. Pharmaceuticals.

[12] Chavada J (2023). Antibiotic resistance: challenges and strategies in combating infections. Cureus.

[13] Littmann, J., *Antibiotic resistance and distributive justice.* 2014.

[14] Banin E, Hughes D, Kuipers OP (2017). Bacterial pathogens, antibiotics and antibiotic resistance. FEMS Microbiol. Rev..

[15] Fatima F, Siddiqui S, Khan WA (2021). Nanoparticles as novel emerging therapeutic antibacterial agents in the antibiotics resistant era. Biol. Trace Element Res..

[16] Govindasamy GA (2021). Effect of calcination temperature on physicochemical and antimicrobial properties of green synthesised ZnO/C/Ca nanocomposites using Calotropis gigantea leaves. Adv. Nat.s Sci.: Nanosci. Nanotechnol..

[17] Jamdagni P, Khatri P, Rana J-S (2018). Green synthesis of zinc oxide nanoparticles using flower extract of Nyctanthes arbor-tristis and their antifungal activity. J. King Saud Univ.-Sci..

[18] Rajeshkumar S (2018). Biosynthesis of zinc oxide nanoparticles usingMangifera indica leaves and evaluation of their antioxidant and cytotoxic properties in lung cancer (A549) cells. Enzyme Microb. Technol..

[19] Stan M (2015). Enhanced photocatalytic degradation properties of zinc oxide nanoparticles synthesized by using plant extracts. Mater. Sci. Semicond. Process..

[20] Govindasamy GA (2023). Giant milkweed plant-based copper oxide nanoparticles for wound dressing application: physicochemical, bactericidal and cytocompatibility profiles. Chem. Papers.

[21] Govindasamy GA (2022). Annealing temperature influences the cytocompatibility, bactericidal and bioactive properties of green synthesised TiO_2_ nanocomposites. Chem. Papers.

[22] Govindasamy GA, Mydin SMN (2023). Phytochemicals, biodegradation, cytocompatibility and wound healing profiles of chitosan film embedded green synthesized antibacterial ZnO/CuO nanocomposite. J. Polym. Environ..

[23] Govindasamy GA (2021). Compositions and antimicrobial properties of binary ZnO–CuO nanocomposites encapsulated calcium and carbon from Calotropis gigantea targeted for skin pathogens. Sci. Rep..

[24] Tran GT (2023). Plant extract-mediated synthesis of aluminum oxide nanoparticles for water treatment and biomedical applications: a review. Environ. Chem. Lett..

[25] Iqbal J (2019). Green synthesis and characterizations of Nickel oxide nanoparticles using leaf extract of Rhamnus virgata and their potential biological applications. Appl. Organ. Chem..

[26] Olajire AA, Mohammed AA (2020). Green synthesis of nickel oxide nanoparticles and studies of their photocatalytic activity in degradation of polyethylene films. Adv. Powder Technol..

[27] Nisar P (2019). Antimicrobial activities of biologically synthesized metal nanoparticles: an insight into the mechanism of action. JBIC J. Biol. Inorg. Chem..

[28] Baranwal A (2018). Prospects of nanostructure materials and their composites as antimicrobial agents. Front. Microbiol..

[29] Wang L, Hu C, Shao L (2017). The antimicrobial activity of nanoparticles: present situation and prospects for the future. Int. J. Nanomed..

[30] Deusenbery C, Wang Y, Shukla A (2021). Recent innovations in bacterial infection detection and treatment. ACS Infect. Dis..

[31] Makarucha AJ, Todorova N, Yarovsky I (2011). Nanomaterials in biological environment: a review of computer modelling studies. Eur. Biophys. J..

[32] Mudshinge SR (2011). Nanoparticles: emerging carriers for drug delivery. Saudi Pharm. J..

[33] Yang W (2021). Nanozymes: activity origin, catalytic mechanism, and biological application. Coord. Chem. Rev..

[34] Weldrick PJ, Hardman MJ, Paunov VN (2019). Enhanced clearing of wound-related pathogenic bacterial biofilms using protease-functionalized antibiotic nanocarriers. ACS Appl. Mater. Interfaces.

[35] Natarajan S, Bajaj HC, Tayade RJ (2018). Recent advances based on the synergetic effect of adsorption for removal of dyes from waste water using photocatalytic process. J. Environ. Sci..

[36] Joseph B, Priya M (2011). Review on nutritional, medicinal and pharmacological properties of guava (Psidium guajava Linn). Int. J. Pharm. Bio Sci..

[37] Azizan NA (2020). Antimicrobial activity of Psidium guajava leaves extract against foodborne pathogens. Int. J. Psych. Rehabilitation.

[38] Dange SS, Rao PS, Jadhav RS (2020). Traditional uses of guava: a review. World J Pharm Res.

[39] Kumari N, Gautam S, Ashutosh C (2013). Psidium guajava a fruit or medicine-An overview. Pharm. Innov..

[40] Flores G (2015). Chemical composition and antioxidant activity of seven cultivars of guava (Psidium guajava) fruits. Food Chem..

[41] Wagatkar, A., et al., *Review on Phytochemicals, Ethnobotanical and Pharmacological Activities of Psidium Guajava.* 2023.

[42] Biswas B (2013). Antimicrobial activities of leaf extracts of guava (Psidium guajava L.) on two gram-negative and gram-positive bacteria. Int. J. Microbiol..

[43] Cáceres A, Cruz SM (2019). Detection and validation of native plants traditionally used as medicine in Guatemala. Curr. Traditional Med..

[44] El-Ahmady SH, Ashour ML, Wink M (2013). Chemical composition and anti-inflammatory activity of the essential oils of Psidium guajava fruits and leaves. J. Essential Oil Res..

[45] Lok B (2020). Anticancer effect of Psidium guajava (Guava) leaf extracts against colorectal cancer through inhibition of angiogenesis. Asian Pacific J. Tropic. Biomed..

[46] Raj A, Menon V, Sharma N (2020). Phytochemical screening, antimicrobial, antioxidant and cytotoxic potential of different extracts of Psidium guajava leaves. Vegetos.

[47] Lim H-W (2020). Antimicrobial activity of Hibiscus sabdariffa L. (Roselle) powder against food-borne pathogens present in dairy products: Preliminary study. J. Dairy Sci. Biotechnol..

[48] Bisht R, Chanyal S, Agrawal PK (2016). Antimicrobial and phytochemical analysis of leaf extract of medicinal fruit plants. Asian J. Pharm. Clin. Res.

[49] Das J, Das MP, Velusamy P (2013). Sesbania grandiflora leaf extract mediated green synthesis of antibacterial silver nanoparticles against selected human pathogens. Spectrochim. Acta Part A: Mol. Biomol. Spectr..

[50] Naik MM (2019). Green synthesis of zinc ferrite nanoparticles in Limonia acidissima juice: characterization and their application as photocatalytic and antibacterial activities. Microchem. J..

[51] Ramadan M, Amin MS, Sayed MA (2020). Superior physico-mechanical, fire resistivity, morphological characteristics and gamma radiation shielding of hardened OPC pastes incorporating ZnFe_2_O_4_ spinel nanoparticles. Constr. Build. Mater..

[52] Nagy Ľ, Zeleňáková A, Hrubovčák P, Barutiak M, Lisnichuk M, Bednarčík J, Vargová J, Jendželovský R, Ševc J, Vilček Š (2023). The role of pH on the preparation of citric acid coated cobalt ferrite nanoparticles for biomedical applications. J. Alloys Compd..

[53] El-Khawaga AM (2020). Evaluation of the antimicrobial activity of citric acid functionalized magnetite nanoparticles. Egypt. J. Med. Microbiol..

[54] Akhlaghi N, Najafpour-Darzi G (2022). Preparation of immobilized lipase on Co_2_+-chelated carboxymethyl cellulose based MnFe_2_O_4_ magnetic nanocomposite particles. Mol. Catal..

[55] Stan M (2016). Antibacterial and antioxidant activities of ZnO nanoparticles synthesized using extracts of Allium sativum, Rosmarinus officinalis and Ocimum basilicum. Acta Metall. Sin. (English Lett.).

[56] Foti MC (2015). Use and abuse of the DPPH radical. J. Agric. Food Chem..

[57] Tiwari S, Mall C, Solanki PP (2022). Evaluation of mixed dye combination by spectral study for the application as photosensitizer in photogalvanic cells for solar energy conversion and storage. Surf. Interfaces.

[58] Maksoud MIAA (2019). Antibacterial, antibiofilm, and photocatalytic activities of metals-substituted spinel cobalt ferrite nanoparticles. Microb. Pathogen..

[59] Khiralla GM, El-Deeb BA (2015). Antimicrobial and antibiofilm effects of selenium nanoparticles on some foodborne pathogens. LWT-Food Sci. Technol..

[60] Stubbings WJ (2004). Assessment of a microplate method for determining the post-antibiotic effect in Staphylococcus aureus and Escherichia coli. J. Antimicrob. Chemotherapy.

[61] Mpangase, S., *An in vitro study of the antimicrobial effect of Indigofera daleiodes plant tinctures using Disc Diffusion and Well Diffusion Assay.* 2020.

[62] Kumar A (2017). Biofilms: survival and defense strategy for pathogens. Int. J. Med. Microbiol..

[63] Azeredo J (2017). Critical review on biofilm methods. Critic. Rev. Microbiol..

[64] Adusei E, Adosraku RK, Oppong-Kyekyeku J, Amengor CD, Jibira Y (2019). Resistance modulation action, time-kill kinetics assay, and inhibition of biofilm formation effects of plumbagin from Plumbago zeylanica Linn. J. Tropical Med..

[65] Mu H (2016). Potent antibacterial nanoparticles against biofilm and intracellular bacteria. Sci. Rep..

[66] Somchaidee P, Tedsree K (2018). Green synthesis of high dispersion and narrow size distribution of zero-valent iron nanoparticles using guava leaf ( Psidium guajava L) extract. Adv. Nat. Sci.: Nanosci. Nanotechnol..

[67] Tholkappiyan R, Vishista K (2014). NN-methylene bis acrylamide: a novel fuel for combustion synthesis of zinc ferrite nanoparticles and studied by X-ray photoelectron spectroscopy. Int J. ChemTech. Res.

[68] Andrade MFC (2016). Lipase immobilized on polydopamine-coated magnetite nanoparticles for biodiesel production from soybean oil. Biofuel Res. J..

[69] Sahoo B, Dutta S, Dhara D (2016). Amine-functionalized magnetic nanoparticles as robust support for immobilization of lipase. J. Chem. Sci..

[70] Mustapha S (2019). Comparative study of crystallite size using Williamson-Hall and Debye-Scherrer plots for ZnO nanoparticles. Adv. Nat. Sci.: Nanosci. Nanotechnol..

[71] Alhadlaq HA, Akhtar MJ, Ahamed M (2015). Zinc ferrite nanoparticle-induced cytotoxicity and oxidative stress in different human cells. Cell Biosci..

[72] Iza, A.M., et al. *Synthesis of zinc ferrite (ZnFe2O4) using microwave assisted coprecipitation method and its effectivity toward photodegradation of malachite green*. AIP Publishing.

[73] Das C (2020). Green synthesis, characterization and application of natural product coated magnetite nanoparticles for wastewater treatment. Nanomaterials.

[74] Das D (2013). Synthesis and evaluation of antioxidant and antibacterial behavior of CuO nanoparticles. Colloids Surf. B: Biointerfaces.

[75] Valerie Siong, L.E., *Green synthesis of reduced graphene oxide for efficient adsorption-photocatalysis studies in methylene blue dye degradation/Valerie Siong Ling Er.* 2020.

[76] Dariani RS (2016). Photocatalytic reaction and degradation of methylene blue on TiO_2_ nano-sized particles. Optik.

[77] Yagub MT, Sen TK, Ang M (2014). Removal of cationic dye methylene blue (MB) from aqueous solution by ground raw and base modified pine cone powder. Environ. Earth Sci..

[78] Nazari Y, Salem S (2019). Efficient photocatalytic methylene blue degradation by Fe_3_O_4_@ TiO_2_ core/shell linked to graphene by aminopropyltrimethoxysilane. Environ. Sci. Pollution Res..

[79] Kragović M (2019). Influence of alginate encapsulation on point of zero charge (pHpzc) and thermodynamic properties of the natural and Fe (III)-modified zeolite. Procedia Manufacturing.

[80] Xu S (2011). Adsorption and photocatalytic degradation of Acid Orange 7 over hydrothermally synthesized mesoporous TiO2 nanotube. Colloids surf. A: Physicochem. Eng. Aspects.

[81] Saleh R, Djaja NF (2014). UV light photocatalytic degradation of organic dyes with Fe-doped ZnO nanoparticles. Superlatt. Microstr..

[82] Zhang P (2017). High efficiency removal of methylene blue using SDS surface-modified ZnFe_2_O_4_ nanoparticles. J. Colloid Interface Sci..

[83] Sawafta R, Shahwan T (2019). A comparative study of the removal of methylene blue by iron nanoparticles from water and water-ethanol solutions. J. Mol. Liquids.

[84] Banerjee P, DasGupta S, De S (2007). Removal of dye from aqueous solution using a combination of advanced oxidation process and nanofiltration. J. Hazard. Mater..

[85] Gong F (2015). An effective heterogeneous iron-based catalyst to activate peroxymonosulfate for organic contaminants removal. Chem. Eng. J..

[86] Reza KM, Kurny ASW, Gulshan F (2016). Photocatalytic degradation of methylene blue by magnetite+ H2O2+ UV process. Int. J. Environ. Sci. Dev..

[87] Maleki A (2010). Study of photochemical and sonochemical processes efficiency for degradation of dyes in aqueous solution. Korean J. Chem. Eng..

[88] Chen Z (2023). Photocatalytic H_2_O_2_ production Systems: design strategies and environmental applications. Chem. Eng. J..

[89] Miranda MO (2021). Photocatalytic degradation of ibuprofen using titanium oxide: insights into the mechanism and preferential attack of radicals. RSC Adv..

[90] Malefane ME, Feleni U, Kuvarega AT (2020). Cobalt (II/III) oxide and tungsten (VI) oxide pn heterojunction photocatalyst for photodegradation of diclofenac sodium under visible light. J. Environ. Chem. Eng..

[91] Das KK (2022). Engineering an oxygen-vacancy-mediated step-scheme charge carrier dynamic coupling WO_3_− X/ZnFe_2_O_4_ heterojunction for robust photo-Fenton-driven levofloxacin detoxification. New J. Chem..

[92] Sriwong T, Matsuda T (2022). Recent advances in enzyme immobilization utilizing nanotechnology for biocatalysis. Org. Process Res. Dev..

[93] Tahir MB (2020). Nanomaterials for photocatalysis. Nanotechnology and photocatalysis for environmental applications.

[94] Torres-Mendieta R (2022). Growth suppression of bacteria by biofilm deterioration using silver nanoparticles with magnetic doping. Nanoscale.

[95] Kumar P, Huo P, Zhang R, Liu B (2019). Antibacterial properties of graphene-based nanomaterials. Nanomaterials.

[96] Abraham W-R (2006). Controlling biofilms of gram-positive pathogenic bacteria. Curr. Med. Chem..

[97] Arciola CR (2012). Biofilm formation in Staphylococcus implant infections. A review of molecular mechanisms and implications for biofilm-resistant materials. Biomaterials.

[98] El-Sayyad GS (2020). Merits of photocatalytic and antimicrobial applications of gamma-irradiated Co x Ni 1–x Fe_2_O_4_/SiO_2_/TiO_2_; x= 0.9 nanocomposite for pyridine removal and pathogenic bacteria/fungi disinfection: implication for wastewater treatment. RSC Adv..

[99] Chatterjee M (2017). Mechanistic understanding of Phenyllactic acid mediated inhibition of quorum sensing and biofilm development in Pseudomonas aeruginosa. Appl. Microbiol. Biotechnol..

[100] Sánchez-López E (2020). Metal-based nanoparticles as antimicrobial agents: an overview. Nanomaterials.

[101] Xiong J, Cai X, Ge J (2022). Enzyme–metal nanocomposites for antibacterial applications. Particuology.

[102] El-Khawaga AM (2023). Promising photocatalytic and antimicrobial activity of novel capsaicin coated cobalt ferrite nanocatalyst. Sci. Rep..

